# Hyaluronic acid microneedle patch for transdermal delivery of naringin-loaded PEGylated terpesomes in a rat model of rheumatoid arthritis: Modulation TGF-β1, oxidative stress, and inflammation

**DOI:** 10.1016/j.ijpx.2026.100527

**Published:** 2026-03-25

**Authors:** Sammar Fathy Elhabal, Ahmed Mohsen Elsaid Hamdan, Mai S. Shoela, Suzan Awad AbdelGhany Morsy, Amal M. Elsharkawy, Rana Saad-eldin, Shady Allam, Tassneim M. Ewedah, Marwa A. Fouad, Mahitab Elsayed, Hanan Mohamed Abd Elmoneim, Halah Tariq Albar, Wedian Younis Abdelgawad, Fatma E. Hassan

**Affiliations:** a Department of Pharmaceutics and Industrial Pharmacy, Faculty of Pharmacy, Modern University for Technology and Information (MTI), Mokattam, Cairo 11571, Egypt; b Department of Pharmacology and Toxicology, Faculty of Pharmacy, University of Tabuk, Tabuk 71491, Saudi Arabia; c Prince Fahad bin Sultan Chair for Biomedical Research (PFSCBR), Tabuk 71491, Saudi Arabia; d Department of Clinical Pharmacology, Faculty of Medicine, Alexandria University, Alexandria 21526, Egypt; e Pathological Sciences Department, MBBS Program, Fakeeh College for Medical Sciences, Jeddah 21461, Saudi Arabia; f Department of Rheumatology and Rehabilitation, Sohag University, Sohag, Egypt; g Department of Pharmacology and Toxicology, Faculty of Pharmacy, Menoufia University, Menoufia, Egypt; h Department of Pharmacology and Toxicology, Faculty of Pharmacy, Menoufia National University, Cairo-Alexandria Agricultural Road, Menoufia, Egypt; i Pharmaceutics and Pharmaceutical Technology Department, Faculty of Pharmacy, Egyptian Russian University, Cairo, Egypt; j Department of Pharmaceutics and Pharmaceutical Technology, Faculty of Pharmacy, Deraya University, Minia 61768, Egypt; k Department of Clinical Pharmacy, Faculty of Pharmacy, Misr International University, Cairo, Egypt; l Department of Pathology, Faculty of Medicine, Umm Al-Qura University, Makkah, Saudi Arabia; m Department of Pathology, Faculty of Medicine, Minia University, Minia, Egypt; n Department of Physiology, Faculty of Medicine, Umm Al-Qura University, Makkah, Saudi Arabia; o Department of Pharmaceutics, Egyptian Drug Authority (Formerly National Organization for Drug Control and Research “NODCAR”), Giza 15301, Egypt; p Department of Physiology, General Medicine Practice Program, Batterjee Medical College, Jeddah 21442, Saudi Arabia; q Medical Physiology Department, Faculty of Medicine, Cairo University, Kasr Alainy, Giza 11562, Egypt

**Keywords:** Transdermal drug delivery, Rheumatoid arthritis, Naringin, Terpesomes, Hyaluronic acid microneedles, TGF-β, Complete Freund's Adjuvant

## Abstract

Rheumatoid arthritis (RA) is a chronic autoimmune inflammatory disease that causes progressive joint damage and systemic effects. Naringin (NRG), a citrus-derived flavonoid with potent anti-inflammatory and antioxidant activities, is hindered by poor solubility and low bioavailability in clinical applications. NRG was encapsulated into PEGylated terpesomes (TPs) prepared by thin-film hydration using phosphatidylcholine, terpenes, and sodium deoxycholate, then integrated into hyaluronic acid–based microneedles (NRG-TPs/HA-MNs) to create a dual-mechanism transdermal delivery system. Optimized NRG-loaded terpesomes possessed a nanoscale particle size (218 ± 0.67 nm), a narrow polydispersity index (PDI: 0.32 ± 0.05), a high entrapment efficiency (79 ± 0.23%), and strong colloidal stability (Zeta potential: −36.37 ± 0.87). HA-MN patches exhibited remarkable mechanical strength, high drug loading (>93%), and successful skin penetration. NRG release was sustained for over 48 h, and transdermal permeation was significantly improved compared to free NRG in both *in vitro* and *ex vivo* studies. In a Complete Freund's Adjuvant–induced RA rat model, NRG-TPs/HA-MNs significantly reduced paw edema and joint swelling, preserved joint architecture on X-ray imaging, and ELISA analysis indicated significant decreases in inflammatory mediators TNF-α, IL-1β, IL-6, NF-κB, and MMP-3. qPCR analysis of the genes MYD88, TXNIP, and BCL-2 showed the therapeutic potential of this system, accompanied by decreased levels of the oxidative stress marker malondialdehyde (MDA), suggesting modulation of the mTOR signaling pathway. Elevated transforming growth factor-β expression and histopathology confirmed cartilage protection and tissue repair. NRG-terpesomal incorporated into the HA-microneedle system provides a minimally invasive therapeutic platform for the sustained release of drugs with improved permeation in the treatment of Rheumatoid arthritis.

## Graphical abstract

**Figure:**
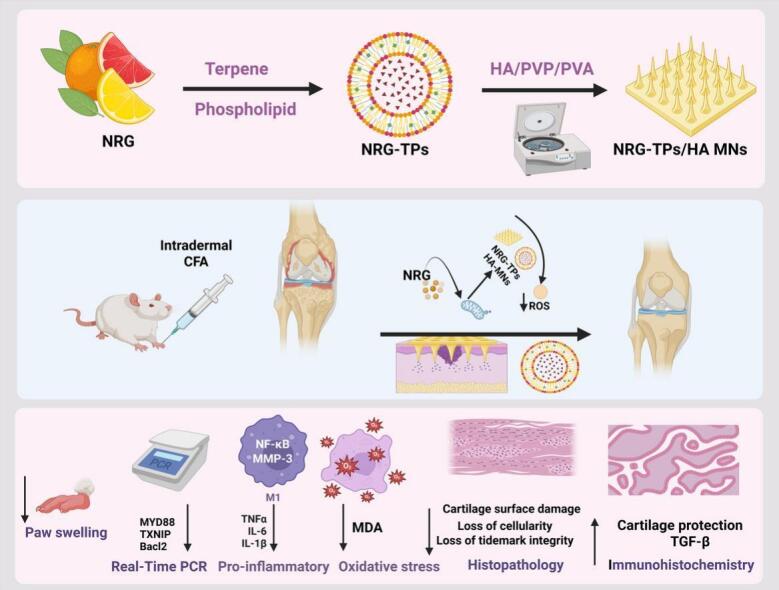

## 1 Introduction

Rheumatoid arthritis (RA) is a chronic autoimmune inflammatory disorder characterized by synovial hyperplasia and progressive joint destruction. Despite advances in pharmacotherapy, conventional treatments such as NSAIDs, corticosteroids, and disease-modifying antirheumatic drugs are associated with systemic side effects and limited patient compliance (Khan et al., 2025b). Therefore, there is a growing need for alternative therapeutic strategies that enhance drug delivery efficiency while minimizing adverse effects (ELhabal et al., 2024; Radu and Bungau, 2021). Synovial membrane hyperplasia leads to irreversible joint deterioration, including cartilage and bone degradation, as well as tendon and ligament injury (Aleith et al., 2025; Zhu et al., 2025). RA symptoms include inflammation, joint swelling, pain, fatigue, and stiffness, which can result in joint impairment, deformity, cartilage and bone destruction, and other health complications (Elhabal et al., 2025b; Yoshitomi, 2019). Researchers have found that IL-1β and TNF-α are essential in cartilage breakdown during arthritis (Díaz-González and Hernández-Hernández, 2023; Yang et al., 2024). RA treatment options include non-steroidal anti-inflammatory drugs (NSAIDs) and methotrexate. To rapidly alleviate symptoms in severely ill patients, procedures such as minimally invasive joint surgery or stem cell transplantation may be employed, alongside disease-modifying antirheumatic drugs, novel biological agents, weight-bearing exercises, medical support, and rest (Cai et al., 2025; Liu et al., 2025a). Medications such as antibiotics and high-dose corticosteroids are used to treat infections and reduce discomfort; however, glucocorticoids can cause serious side effects, including bone loss and hyperglycemia (Elhabal et al., 2025b; Martínez-Ramos et al., 2025). Early management of pain and disease progression often involves long-term oral or intra-articular medication. Traditional Chinese medicine techniques, such as herbal decoctions, acupuncture, and moxibustion, can complement pharmaceutical treatments (ELhabal et al., 2024; Zhou et al., 2025).

Citrus flavonoids, a diverse group of flavonoids, exhibit anti-inflammatory properties, decrease lipid peroxidation (LPO), and enhance the antioxidant defense system (Ref). The antioxidant properties of the peel (flavedo and albedo) and juice of commercially cultivated citrus fruits (Rutaceae), specifically grapefruit (Citrus paradisi), lemon (*Citrus limon*), lime (*Citrus aurantiifolia*), and sweet orange (*Citrus sinensis*), were evaluated *in vitro* (Nazir et al., 2025; Vasava et al., 2026). Studies on the peels and juices of common citrus fruits revealed that their components can neutralize free radicals and prevent lipid peroxidation. Flavonoids, including naringin, which is abundant in grapefruit (Vasava et al., 2026). It is known for several biological effects, including the protective effects on the liver, heart, and nerves; anti-inflammatory, antioxidant, cholesterol-lowering, and anti-cancer effects. Naringin has caused cell death in several cancer cell lines while leaving normal cells unharmed, and its potential has been investigated in rheumatoid arthritis (RA) and osteoporosis, where it seems to play a role in bone formation and stem cell differentiation (Nazir et al., 2025; Saleem et al., 2025).

Naringin (NRG), a bioactive, water-insoluble glycoside, is a flavonoid widely found in grapefruit, citrus fruits, bergamot, oregano, and tomatoes, all of which are known to be rich in NAR. The compound has exhibited a variety of pharmacological activities, including hepatoprotective, anti-inflammatory, antioxidant, antiatherogenic, neuroprotective, hypocholesterolemic, cardioprotective, antidiabetic, osteogenic, and anticancer effects in thyroid, prostate, breast, cervical, and colon cancer cell lines, without showing inherent toxicity (Chen et al., 2025a). NRG has anticancer effects by selectively increasing cytotoxicity and apoptosis in cancer cells, but not in normal cells (Ahmed et al., 2019; Ahmed et al., 2024). The therapeutic effects and mechanisms of NAR, a naringenin-derived flavanone glycoside, in RA were confirmed through *in vitro* and clinical validation, as well as network pharmacology analysis (Aihaiti et al., 2021). Naringin is used in traditional Chinese medicine to treat osteoporosis. NRG can promote cellular signaling pathways important for bone and cartilage formation. The effects of naringin on bone health include its potential to treat osteoporosis and promote osteogenic differentiation of mesenchymal stem cells (MSCs). Naringin has other beneficial effects in tissue engineering and therapeutic potential due to its pharmacological actions on intracellular targets. NRG has been documented to decrease clinical symptom severity and levels of inflammatory mediators and proinflammatory cytokines in multiple *in vitro* and *in vivo* studies (Chen et al., 2025b; Dar et al., 2024; Shaaban et al., 2022). The biological activity of NRG is affected by the low water solubility (0.475 mg/mL at 25 °C), poor bioavailability and permeability, and poor systemic absorption due to significant first-pass metabolism. Moreover, the intestinal environment has been reported to be unstable in the presence of NRG. While the β-cyclodextrin-coated NRG formulation exhibited approximately a 15-fold higher water solubility than free NRG, it caused gastrointestinal irritation upon oral administration. The therapeutic use of NRG can be more efficient by combining individual nanocarriers with surface modification strategies that enhance water stability. Nevertheless, effective pharmacological approaches are fundamental in the treatment of RA. Conventional therapies, in combination with novel biological agents, have significant limitations: gastrointestinal discomfort and low absorption are consequences of systemic oral pharmacological therapies. There are also poor patient compliance and increased risk of infection, which can be serious complications in non-therapeutic target tissues, due to intra-articular injections. These limitations contribute to the stagnation in advances in rheumatoid arthritis treatment (Akanksha et al., 2025; Khan et al., 2025a; Lai et al., 2025).

In recent years, Transdermal Drug Delivery Systems (TDDS) have been developed and marketed in the health industry. These systems have developed competitive advantages over oral systems and systems that provide international administration, as they do not expose patients to over-elimination and do provide transmucosal effects to mitigate. Despite these competitive advantages, the body's largest barrier, the skin, has perniciously slow passive diffusion. Therefore, the progressive identification and development of the means to transport Terpesomes across the skin barrier to the targeted location remains profoundly significant in the field of transdermal systems (Ananda et al., 2021; Babaie et al., 2020).

Terpenes are natural compounds developed to enhance the skin-penetration of biosourced materials. With these, the first Ultra-Deformable Vesicular System (UDVS) termed \”Terpesomes\” was developed (Dwivedi et al., 2024). Terpesomes are vesicular nanostructures composed of phospholipids, a single-chain, low-molecular-weight alcohol (ethanol), and one or several terpenes. In Terpesomes, the phospholipid is the primary bilayer matrix, and the terpenes and ethanol incorporated into the bilayer and the vesicular wall provide fluidity and alter the molecular structure of the bilayer (Rossi et al., 2023; Saffari et al., 2016). Terpenes are a ubiquitous and diverse group of plant-based compounds that can enhance the encapsulation and transport of a broad spectrum of pharmaceuticals, thereby increasing their bioavailability. Even more interesting, some terpenes have been shown to have hepatoprotective effects; therefore, Terpesomes are the optimal carriers for delivering hepatoprotective pharmaceuticals. Importantly, PEGylation, the process of attaching PEG (polyethylene glycol) chains to molecules or nanoparticles, increases nanoparticle stability. Also, PEGylation reduces immunogenicity and increases circulation time due to increased hydrophilicity, reduced renal clearance, and a steric barrier to enzymatic degradation (Elhabal et al., 2025f). Furthermore, it decreases immunogenicity and improves blood circulation time by increasing hydrophilicity, decreasing renal clearance, and providing a steric barrier to enzymatic breakdown.

The microneedle (MN) platform offers a transdermal method of medication delivery. It has become widely used in recent years as a drug carrier for various conditions, including cancer, diabetes, and psoriasis; for vaccine delivery; and for measuring physiological indicators. MNs can be classified as solid, coated, hollow, dissolving, or hydrogel types, depending on the tip material and drug distribution method (Elhabal et al., 2025d; Elhabal et al., 2025a; Moon et al., 2026). Dissolving MNs can effectively penetrate the skin's stratum corneum and enhance drug transport, making them a prominent focus of TDDS research recently. They can breach the skin, form drug-delivery channels, and avoid contact with nerves and blood vessels in the dermis, which reduces discomfort and the risk of infection (Sartawi et al., 2022; Stoeva et al., 2025). Compared to other metal-based MNs, dissolving MNs have a higher drug-carrying capacity and can dissolve to release loaded pharmaceuticals within the skin, thus minimizing medical waste.Microneedle-based systems have also shown varying degrees of effectiveness in biomedically useful functions beyond just transdermal administration. These functions include advanced localized therapy, immunomodulation, and regenerative medicine. A recently published example includes a microneedle platform for near-infrared responsive immunomodulation, and for accelerated chronic wound healing, inspired by mesenchymal stem cells. Another example is a detachable microneedle patch developed for the localized delivery of a TGF-β inhibitor to diminish scar tissue. These investigations also emphasize the versatile and rapid promise of microneedle-assisted delivery in various therapeutic biomedical applications (Moon et al., 2026; Park et al., 2025).

The present study develops and evaluates a novel transdermal system to enhance the therapeutic efficacy of naringin (NRG) in rheumatoid arthritis (RA). PEGylated terpesomal nanocarriers incorporating penetration-enhancing terpenes are embedded in hyaluronic acid microneedle patches (HA-MNs), which dissolve upon insertion to enable dual-mechanism cutaneous delivery. A D-optimal design optimizes NRG-loaded terpesomes with respect to phosphatidylcholine type and concentration and their impact on particle size, homogeneity, zeta potential, and entrapment efficiency. The optimized formulations are characterized by TEM, FTIR, DSC, and stability testing, then integrated into two-step cast HA-microneedles yielding strong, dissolvable patches with adequate drug content and robust mechano-inserts. *In vitro* release and *ex vivo* penetration studies in rat skin compare the system with free NRG, confirming prolonged release and enhanced transdermal delivery. In a rat RA model induced by Complete Freund's Adjuvant, therapeutic efficacy is assessed *via* paw volume and diameter, radiography, inflammatory markers (TNF-α, IL-6, NF-κB, IL-1β, MMP-3), oxidative stress (MDA), and regulatory proteins (mTOR, TGF-β), supported by histopathology and immunohistochemistry. The designed system aims to improve NRG bioavailability, achieve sustained release, suppress inflammation, and enhance overall therapeutic outcomes in RA through a non-invasive, user-friendly transdermal platform.

## 2 Materials and methods

### 2.1 Chemicals and reagents

Naringin (purity >95%) (NAR) (4′,5,7-trihydroxyflavanone 7-rhamnoglucoside; C_27_H_32_O_14_) and L-α phosphatidylcholine derived from soybean oil (PC) were purchased from Sigma-Aldrich Co. (Saint Louis, MO, USA). Terpenes, including fenchone, cineole, and limonene, along with sodium deoxycholate (SDC) and sodium dithionite, were acquired from Alfa Aesar (GmbH, Germany). Complete Freund's adjuvant (CFA) was obtained from Santa Cruz Biotechnology, Inc., in Texas, USA. Hydroxypropyl methylcellulose (HPMC; K4M) was supplied by Dow Chemical Company in Midland, MI. Disodium hydrogen phosphate, sodium chloride, potassium dihydrogen phosphate, ethanol, methanol, and chloroform were purchased from El-Nasr Pharmaceutical Chemicals Co. in Cairo, Egypt. Spectra Por® dialysis membrane tubing with a molecular weight cutoff of 14 g/mol was obtained from Spectrum Laboratories Inc. (California, USA). All other chemicals were used in their original forms.

### 2.2 Animals

Fifty adults male Wistar rats, aged 6 weeks and weighing between 170 and 190 g, were obtained from the animal breeding facility at the National Research Centre in Egypt. The animals were housed in plastic cages for one week under controlled conditions, including a 12-h light/dark cycle, a temperature of 24 ± 1 °C, and a relative humidity of 55%. The subjects were provided with a standardized purified diet, AIN-93, as required. They had unrestricted access to food and water. All procedures were carried out in accordance with established protocols, and the committee's policies comply with the National Institutes of Health Guidelines for the Care and Use of Laboratory Animals and the ARRIVE guidelines (MD, USA). We followed the procedures outlined in the Guide for the Care and Use of Laboratory Animals, published by the US National Institutes of Health (NIH Publication No. 85–23, revised 2011). The Institutional Research Ethics Committee (REC) at the Faculty of Pharmacy, Cairo University, approved this study, with the approval number (PI 3999).

## 3 Methods

### 3.1 Naringin high-performance liquid chromatography analysis

A method for measuring naringin was developed using high-performance liquid chromatography (HPLC). The analysis used a Shimadzu DGU-20 As system comprising an LC-20 AT liquid chromatography pump, an SPD-M20A UV/VIS detector, and a SIL-20A HT autosampler. A C-18 column (25 cm × 4.6 mm, 5 μm) was used for separation. C18 combines a silica surface grafted with 18‑carbon alkyl chains, further increasing hydrophobicity, retention, and selectivity. The mobile phase was a 30:70 (*v*/v) mixture of acetonitrile and water, at 1.0 mL/min under isocratic conditions. NRG's max absorbance was at 282 nm, so detection was set to 282 nm. The method demonstrated a linear relationship (R^2^ = 0.999)(Büyüktuncel, 2017; Chavda et al., 2025; Moon et al., 2014). To maintain sink conditions and avoid external auto-concentration, at regular time intervals, 300 μL samples were taken, and pre-warmed phosphate-buffered saline (PBS, pH 7.4)was added to replace the sink.

### 3.2 Experimental design Naringin-loaded Terpesomes (NRG-TPs)

For NRG Terpesomes, a D-optimal design was used (3^3^). The three factors were the amount of PC (mg) (A), the terpene concentration (%*w*/*v*) (B), and the terpene type (C). Based on preliminary tests, levels −1, 0, and + 1 were set. The four responses were size (Y1), polydispersity (Y2), zeta potential (Y3), and entrapment efficiency percentage (EE%; Y4), which were the main factors of significance. These were performed using Design-Expert® (Stat-Ease, Inc., Minneapolis, MN, USA). The actual values of the selected parameters and criteria for the optimal formulation are shown in Table 1.

**Table 1:** D-Optimal factorial design utilized for optimizing NRG-TPs formulations.

Factors (Independent variables)	Design Levels		
	Low (−1)	Medium (0)	High (+1)
A: PC amount (mg)	80	120	160
B: Terpene conc. (%w/v)	1	2	4
C: Terpene type	limonene	fenchone	Cineol
Responses (Dependent variables)	Goal		
Y1: P.S. (nm)	Minimize		
Y2: PDI (nm)	Minimize		
Y3: Z.P (mV)	Maximize		
Y4: E.E (%)	Maximize		

Abbreviations: PC, Phosphatidylcholine; P.S., Particle size; PDI, polydispersity index; ZP, zeta potential, and E.E., entrapment efficiency.

### 3.3 Preparation of Naringin-loaded Terpesomes (NRG-TPs)

**Fig. 1:**
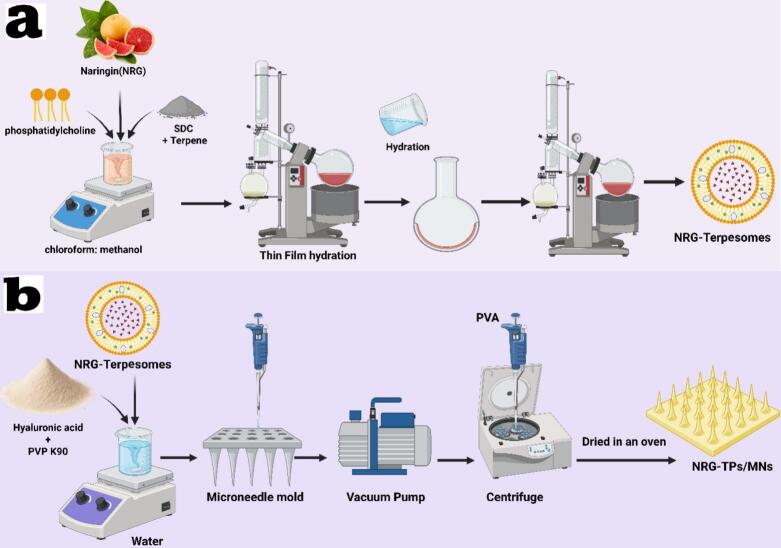
(a)Diagram illustrates the methodology for preparing Naringin-loaded Terpesomes (NRG-TPs), and (b) Diagram illustrates the methodology for fabrication of multifunctional Optimized NRG-TPs-loaded HA microneedle patches (NRG-TPs/HAMNs).

The thin film hydration method was used to prepare twenty-four NRG-TPs with four different variables: (A) the amount of PC (80, 120, or 160 mg), (B) the type of terpene (fenchone, cineole, or limonene), (C) the concentration of terpene (1%, 2%, or 4% w/v), and (D) the concentration of sodium deoxycholate (SDC) (0.1% *w*/*v*). First, NRG (10 mg), PC, terpene, and SDC (0.1%) were dissolved in a mixture of chloroform and methanol (2:1) in a flask using sonication (Crest Ultrasonics Corp., Trenton, USA), as shown in Table 2. The organic mixture was extracted, producing a thin, clear layer of terpesomes by maintaining reduced pressure at 120 rpm and 60 °C for 30 min using a rotary evaporator (Heidolph VV 2000, Burladingen, Germany). Film hydration was effectively achieved by adding the hydrating medium (10 mL of 3% *v*/v ethanolic distilled water), rotating the flask at 120 rpm at ambient pressure and room temperature for 30 min, and adding small glass beads (Mahdy et al., 2025; Zarif Attalla et al., 2025). The terpesomal dispersions (final NRG concentration: 2 mg/mL) were stored overnight at 4 °C to allow for equilibration and maturation, as shown in Fig. 1a.

**Table 2:** Composition of NRG-loaded Terpesomes with their measured responses (*n* = 3 ± SD).

Run	Factors			Responses			
	A: PC amount (mg)	B: Terpene conc (w/v%)	C: Terpene type	P.S. (nm)	PDI (nm)	Z.P (mV)	EE (%)
1	120	4	limonene	181 ± 0.45	0.31 ± 0.09	−38 ± 0.39	89 ± 0.93
2	120	2	fenchone	390 ± 0.87	0.46 ± 0.07	−26 ± 0.65	58 ± 0.85
3	160	4	fenchone	435 ± 0.67	0.49 ± 0.06	−29 ± 0.63	61 ± 0.67
4	160	1	fenchone	450 ± 0.48	0.52 ± 0.03	−28 ± 0.56	57 ± 0.88
5	80	1	fenchone	390 ± 0.78	0.51 ± 0.05	−25 ± 0.57	56 ± 0.78
6	120	1	cineole	440 ± 0.95	0.54 ± 0.06	−35 ± 0.87	71 ± 0.57
7	160	4	limonene	215 ± 0.98	0.35 ± 0.02	−38 ± 0.92	83 ± 0.68
8	160	4	cineole	510 ± 0.68	0.41 ± 0.06	−35 ± 0.67	62 ± 0.76
9	120	1	limonene	230 ± 0.64	0.39 ± 0.04	−37 ± 0.65	80 ± 0.56
10	120	4	cineole	490 ± 0.87	0.48 ± 0.03	−36 ± 0.34	60 ± 0.77
11	80	2	cineole	530 ± 0.85	0.39 ± 0.04	−39 ± 0.65	59 ± 0.67
12	160	2	cineole	430 ± 0.56	0.34 ± 0.02	−31 ± 0.66	56 ± 0.95
13	80	4	limonene	310 ± 0.45	0.35 ± 0.01	−35 ± 0.56	76 ± 0.65
14	120	4	fenchone	320 ± 0.63	0.54 ± 0.04	−31 ± 0.34	55 ± 0.45
15	120	2	fenchone	355 ± 0.56	0.52 ± 0.05	−27 ± 0.65	61 ± 0.65
16	120	2	fenchone	310 ± 0.77	0.45 ± 0.06	−24 ± 0.56	65 ± 0.67
17	80	2	limonene	420 ± 0.98	0.33 ± 0.03	−33 ± 0.66	68 ± 0.43
18	80	2	cineole	550 ± 0.96	0.34 ± 0.05	−32 ± 0.76	57 ± 0.76
19	120	4	cineole	450 ± 0.95	0.55 ± 0.03	−33 ± 0.45	67 ± 0.56
20	80	4	fenchone	360 ± 0.67	0.56 ± 0.07	−27 ± 0.66	66 ± 0.66
21	160	2	limonene	250 ± 0.56	0.34 ± 0.02	−37 ± 0.34	78 ± 0.35
22	120	2	fenchone	320 ± 0.89	0.56 ± 0.04	−32 ± 0.86	59 ± 0.86
O. F	130	3	limonene	218 ± 0.67	0.32 ± 0.05	−36.37 ± 0.87	79 ± 0.23

Abbreviations: PC, Phosphatidylcholine; P.S., Particle size; PDI, polydispersity index; ZP, zeta potential; E.E., entrapment efficiency; and O.F., optimized formula.

### 3.4 *In-vitro* characterization of NRG-loaded terpesomes (NRG-TPs)

#### 3.4.1 Hydrodynamic diameter analysis of particle size (PS), polydispersity index (PDI), and zeta potential (ZP)

The average particle size of NRG-loaded terpesomes was measured at 25 ± 1 °C using photon correlation spectroscopy on a Malvern Zetasizer Nano ZS (Worcestershire, UK) following a 15-fold dilution with deionized distilled water. The PDI values served to evaluate the uniformity of the particle size distribution. The ZP values of negatively charged terpesomal vesicles were determined by assessing their electrophoretic mobility with the same equipment (Elhabal et al., 2025c, Elhabal et al., 2025e).

#### 3.4.2 Determination of NRG entrapment efficiency (EE%)

The entrapment efficiency (EE%) of NRG was assessed using a non-destructive spectroscopic method with a Shimadzu UV-1601 PC (Kyoto, Japan) to measure the free NRG in the supernatant. The optimized preparation was centrifuged at 21,000 rpm for 1 h at 4 °C (Al-Zuhairy et al., 2025; Youssif et al., 2025). The diluted supernatant was analyzed *via* High-Performance Liquid Chromatography (HPLC) employing a UV–Vis detector (Agilent 1260 Infinity II/DAD, Agilent Technologies, Santa Clara, USA) to determine the concentration of unentrapped NRG at 282 nm (*n* = 3; R2 = 0.9997). The EE% was calculated using the formula (Fathy Elhabal et al., 2026; Elhabal et al., 2025a, Elhabal et al., 2025b, Elhabal et al., 2025c, Elhabal et al., 2025d, Elhabal et al., 2025e, Elhabal et al., 2025f, Elhabal et al., 2025g): EE% = × 100 (Eq. 1).

(1) $$\mathrm{EE}\%=\frac{\text{Total}\;\mathrm{NRG}\;\text{concentration}-\text{Concentration of unentrapped}\;\mathrm{NRG}\;}{\text{Total}\;\mathrm{NRG}\;\text{concentration}}X\;100$$

#### 3.4.3 Optimizing NRG-TPs

Using Design-Expert® software, a D-optimal design was used to analyze the effects of variables on the reactions of the generated Terpesomes. To find the optimal formula (with the highest attractiveness), the absolute values of ZP and EE% were maximized, while PDI and particle size were minimized. This approach employs a numerical optimization technique to select the formula with the highest value (approximately 1). The ideal formula underwent extensive physicochemical, *in vivo*, and *ex vivo* evaluations to confirm its safety, stability, and efficacy (El-Emam et al., 2021; Salvi and Shende, 2025).

#### 3.4.4 Physicochemical characterization of the optimum formula

##### 3.4.4.1 Transmission electron microscopy (TEM)

The optimal formulation was diluted with double-distilled water, applied to carbon-coated copper grids, and stained with 2% phosphotungstic acid. The morphological properties were analyzed using a transmission electron microscope (TEM; JEOL, Tokyo, Japan) (Elhabal et al., 2025g; Ricci et al., 2024).

##### 3.4.4.2 Fourier transform infrared spectroscopy (FTIR) studies

An FTIR spectrophotometer (model 22, Bruker, Coventry, UK) was used to identify the FTIR peaks of NRG, Blank TPs, and the optimal formula NRG-TPs. FTIR helped to detect possible interactions between the constituents and confirm the entrapment of NRG within the terpesomes produced. The materials were dehydrated and then pressed into KBr discs. All samples were analyzed at 25 °C across the spectral range 4000–500 cm^−1^ (Fayyaz et al., 2025; Hendawy et al., 2025).

##### 3.4.4.3 Crystallinity examination via differential scanning calorimetry (DSC) studies

NRG-loaded terpesomes were generated, frozen, and lyophilized for 24 h at −45 °C using a Novalyphe-NL 500 lyophilizer (Savant Instruments, NY, USA) under a pressure of 7 × 10–2 mbar. The thermal properties of optimal lyophilized terpesomes, the NRG, the physical mixture of all solid components of Blank TPs, and the optimal formula NRG-TPs were analyzed using a Shimadzu differential scanning calorimeter (DSC-60 Shimadzu, Kyoto, Japan). The samples underwent scanning in aluminum pans across a temperature spectrum of 30–300 °C, utilizing a heating rate of 10 °C/min while being subjected to a nitrogen stream at 20 mL/min, with indium serving as the reference point (Mohamed et al., 2025; Zafar et al., 2023).

##### 3.4.4.4 Stability study

The optimized formulae were maintained at 4–8 °C and at ambient temperature (20 ± 5 °C) for periods of 3 and 6 months, respectively, with stability assessments conducted to evaluate their quality and activity. The physical appearance, particle size (PS), polydispersity index (PDI), entrapment efficiency (EE%), and zeta potential (ZP) were analyzed and compared with those of samples prepared after 24 h (Issuriya et al., 2025; Wang et al., 2013). A one-way ANOVA was conducted to compare EE%, PS, PDI, and ZP. A similarity factor “ƒ2” was employed to analyze release profiles through the following equation (Eq. 2):

(2) $$ƒ2=50.\log\{\left[1+\left(\frac{1}{n}\right)\;\sum_{t=1\;}^{n}\left(R\;t-\mathrm{Tt}\right)\;2\right]-0.5.100$$

#### 3.4.5 Fabrication of hyaluronic acid microneedle patches

The NRG-TP-loaded HA MNs was fabricated using a 1 cm × 1 cm PDMS mold (Silicone Template ST-01; Micropoint Technologies Pte Ltd., Singapore). The MNs had a conical shape, with a rounded bottom, arranged in a 10 × 10 pattern of holes measuring 300 μm in height, 100 μm in basal diameter, and 5 μm at the tip. The NRG-TP-loaded HA MNs were separated into two layers: the top layer containing the medication and the base layer. To prepare the needle tip gel solution shown in Table 3, dissolve 600 μL of NRG-TPs in 0.5 mL of anhydrous ethanol, then add HA at concentrations of 100, 200, or 300 mg, along with 50, 100, or 200 mg of PVP K90. Mix vigorously to ensure polymer crosslinking (Elhabal et al., 2025b; Nagra et al., 2022). A two-step casting process was used to produce the NRG-TP-loaded HA MNs. The tip solution was applied to the mold surface and defoamed with a vacuum pump (VALUE, CH11871, China) to ensure it entered the voids and filled the mold; excess tip solution was scraped off. Next, a 15% *w*/*v* PVA gel solution was added to form the substrate layer of the MNs by centrifuging at 3000 rpm for 5 min. Finally, the drug-filled molds were baked in an oven at 37–40 °C. The NRG-TP-loaded HA MNs was then carefully removed and stored dry at room temperature (Fig. 1b).

**Table 3:** Fabrication of HA-MNs loading freeze-dried Naringin-loaded Terpesomes (NRG-TPs).

Formulations	Hyaluronic acid (mg)	PVP K90
HA-MNs1	100	200
HA-MNs1	200	100
HA-MNs1	300	50

#### 3.4.6 Characterization of microneedle patches

##### 3.4.6.1 Drug contents

Magnetic agitators comprising NRG-TPs/HA-MNs were dissolved in distilled water with 2.5% Tween 80 and agitated at 300 rpm for one hour. The solution was diluted with methanol and sonicated for 5 min to ensure complete dissolution of NRG within the NRG-TPs/HA-MNs. The validated HPLC method described in the previous section was used to perform this procedure. The drug content of the produced HA-MNs was then validated (Stoeva et al., 2025; Wang et al., 2025b).

##### 3.4.6.2 Mechanical strength and penetration capability test

Weights of 250, 500, and 1000 g were applied to the tips of the NRG-TPs/HA-MNs patch for five minutes before removal to evaluate the reduction in DMN height. The DMN was evaluated by optical microscopy and fracture analysis, a method for assessing mechanical strength. Parafilm M® (Bemis Company Inc., USA), a flexible olefin-based thermoplastic film commonly used to replicate the mechanical properties of human skin, was employed following Elhabal's notably revised methodology (Elhabal et al., 2025c; Shabbir et al., 2022). A film composed of eight layers was folded to mimic the manual insertion of microneedles (HA-MNs) into the skin. MNs arrays were subsequently applied to the film with a force of 32 N and a speed of 0.1 mm/s. The array was subsequently retrieved from the Parafilm with precision. After the Parafilm layers were removed, the holes created by insertion were measured using a C-B10+ stereomicroscope (Optika, Italy) and Eq. (3). These measurements enhance the structural strength of microneedles under force and their effectiveness in penetrating skin-like materials.

(3) $$\%\text{Penetration capability}=\left(\text{Number of holes}\right)/\left(\text{Total needles}\right)\times 100\%$$

#### 3.4.7 Characterization of optimized microneedle

##### 3.4.7.1 Scanning electron microscopy (SEM)

Scanning electron microscopy (SEM) is a technique that uses a focused electron beam to generate high-resolution images of surfaces. This approach facilitates the study of the topography and composition of materials at the micro- and nanoscale. The morphology of the NRG-TPs/HA-MNs patch was studied with a scanning electron microscope (SU8010, Hitachi, Japan). The NRG patch was mounted on a microscope carriage, and imaging was performed at 6 kV with 150× magnification (Costa et al., 2025; de Souza et al., 2025; Elhabal et al., 2024a).

##### 3.4.7.2 Fourier transform infrared (FTIR) analysis

An FTIR spectrometer was used to record the room-temperature spectra of an NRG-TPs/HA-MNs patch and its constituent parts between 4000 and 500 cm^−1^ (model 22, Bruker, Coventry, UK).

##### 3.4.7.3 Differential scanning calorimetry (DSC)

The thermal properties, crystallinity, and interactions among the NRG-TPs/HA-MNs patch were examined using differential scanning calorimetry (DSC-60; Shimadzu, Kyoto, Japan) at temperatures from 25 to 250 °C, with nitrogen purging at 100 ml/min, as described previously (Elhabal et al., 2024a).

##### 3.4.7.4 Drug release studies

###### 3.4.7.4.1 In vitro drug release study

To evaluate NRG release behavior after loading into NRG-TPs, NRG-TPs/HA-MNs, and free NRG solution (1 mg/mL in 25% PEG 400), samples were placed in VISKING® 36/32 dialysis bags (28 mm, MWCO 12,000-14,000; Serva, Heidelberg, Germany) and immersed in 12 mL of 25% *v*/v PEG 400 in PBS (pH 7.4) as a release medium. To maintain sink conditions, the setup was maintained at 37 °C with shaking at 100 rpm (Alhallak et al., 2023; Fan et al., 2025). Samples were collected at specified time points and analyzed by HPLC at 282 nm, with the release medium serving as the blank. To ensure consistent sink conditions, withdrawn samples were replaced with fresh release medium (Elhabal et al., 2025e). The total amount of NRG released from each formulation was calculated, corrected, and expressed as a percentage of the total drug released over time, using Eq. (4).

(4) $$\%\text{Cumulative drug release}=\frac{\text{Sample volume taken}\;\left(\mathrm{ml}\right)}{\;\text{Bath volume}\;\left(v\right)}\;x\;P\left(t-1\right)+\mathrm{Pt}$$

where Pt is the percentage of the amount released at the time (t) and P(t-1) is the percentage of the amount released per time (t).

Kinetic analysis of NRG release from NRG suspension, optimal NRG-TPs, and NRG-TPs/HA-MNs was conducted using zero-order and first-order models. The model with the highest R^2^ (*i.e.*, the linear model) was selected. Each release mechanism was also evaluated with the Higuchi diffusion and Korsmeyer-Peppas models. Mathematical modeling and calculations were performed in Microsoft Excel (Microsoft Office 365, One Microsoft Way, Redmond, WA, USA), and R^2^ values were determined for each model. The zero-order model was represented by a plot of (Qn) *versus* (time), while the first-order model was shown as a plot of (log % remaining [log (100 - Qn)]) *versus* (time) [64]. The Higuchi diffusion pattern was displayed as (Qn) *versus* (√ time). For Korsmeyer-Peppas, (log Qn) was plotted against (log time), and the release exponent (n) was calculated to understand how NRG was released from the NRG suspension, the optimal NRG-TPs formulation, and NRG-TPs/HA-MNs.

The following equations describe the applicable mathematical models (Ahmadi et al., 2025; Salama et al., 2022):

Zero order:

(5) $$Q_{n}=Q_{0}+k_{0}t$$

First-order kinetics:

(6) $$\mathrm{Log}\;\left(100-\mathrm{Qn}\right)=\log\;Q0-\frac{\mathrm{Kt}}{2.303}$$

Higuchi diffusion:

(7) $$Q_{n}=k_{H}\surd t$$

Korsmeyer-Peppas:

(8) $$\mathrm{Log}\;Q_{n}=\log k+n\text{logt}$$

##### 3.4.7.5 Ex vivo permeation studies

*Ex vivo* transdermal permeation tests on rat skin were conducted using NRG suspension, the optimized NRG-TPs formulation, and NRG-TPs/HA-MNs Franz diffusion cells with an orifice diameter of 9 mm, equivalent to 0.64 cm of skin area. The skin was excised to the same thickness and carefully shaved with a razor. The acquired skin was stored at 4 °C until needed. The skin was pre-equilibrated in the appropriate PBS (pH 7.4) for 30 min before experimentation. To ensure complete contact between the fluid in the receiver compartment and the rat's skin, degassed PBS (pH 7.4) was delivered to the individual receiver compartment and preheated to 37 °C (Qaiser et al., 2024; Zhang et al., 2024). The NRG suspension, the ideal NRG-TPs formulation, and the NRG-TPs/HA-MNs were each put into the skin samples using firm finger pressure for 30 s. Immediately, the MN-inserted skins were sandwiched horizontally between the clamped donor and receptor compartments. The stratum corneum was adjacent to the donor chamber, whereas the subcutaneous skin tissues faced the receptor chamber (Ewedah et al., 2024). Later, parafilm was used to seal the donor chamber. As a control group, equal volumes of NRG suspension, the optimal NRG-TPs formulation, and NRG-TPs/HA-MNs were mixed in solutions and added to donor compartments of different Franz diffusion cells. To maintain sink conditions, 200 L aliquots were withdrawn from the receptor medium using a microsyringe at predetermined intervals (up to 24 h) and replaced with an equivalent volume of fresh, preheated medium (ELhabal et al., 2024b). The Franz cells were incubated in a water bath at 37 °C, and the receptor medium was continuously stirred. Later, with appropriately diluted samples, NRG concentrations were estimated using the same method, and the percentage drug permeation was plotted against time (Teixeira et al., 2024).

### 3.5 In vivo study

#### 3.5.1 Induction of rheumatoid arthritis in rats

**Fig. 2:**
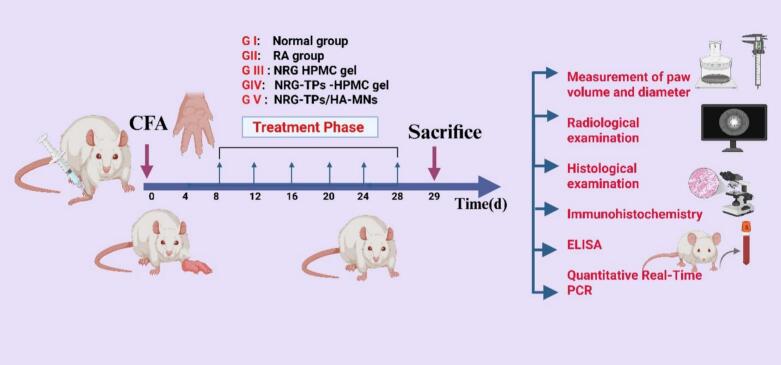
Schematic of the *in vivo* experiment. CFA, complete Freund's adjuvant.

The Rheumatoid Arthritis model was established using male Sprague-Dawley rats, intradermal injection into the right hind metatarsal footpad with 0.1 mL of Complete Freund's Adjuvant (CFA) (10 mg/mL) on day 0, which resulted in significant swelling of the right hind paw, on 8th day following RA generation (ELhabal et al., 2024; Liang et al., 2025), Fifty Rats were randomly assigned to five groups (*n* = 10): Group I (healthy negative control group; act as control), Group II (RA model group, untreated), Group III (RA treated with NRG incorporated in gel containing 2% (*w*/w) HPMC), Group IV (RA treated with optimal NRG-TPs incorporated in gel containing 2% (w/w) HPMC), The gel (1 g) was applied on the knee joint surface of each test animal to an area of 4 cm^2^ and Group V (RA treated with optimal NRG-TPs /HA MNs), for administration in the MNs group, the MNs were inserted with the thumb into the prepared thigh hairless skin at the non-acupuncture points and fixed with medical tape for 20 min to ensure that the needles were wholly dissolved. All formulations are equivalent to 20 mg/kg NRG. The arrays were replaced every third day for 3 weeks, as illustrated in Fig. 2. The rats were quickly killed by cervical dislocation as soon as the blood was retrieved. The right knee joint, including the distal femur and proximal tibia, was dissected and preserved in neutral buffered formalin at a 10% dilution for 72 h. Knee joint samples were meticulously separated from the surrounding soft tissue and muscles. For four weeks, each experimental group of rats was decalcified with a weekly change of a 10% ethylenediaminetetraacetic acid (EDTA) solution at pH 7.4, buffered at 4 °C. Knee joints were processed for histological pathological examination.

#### 3.5.2 Measurement of Paw volume and paw diameter

Paw volume and diameter are indicators of swelling due to inflammation and serve as direct markers of RA. The volume of the RA-induced left hind paw was measured using a calibrated plethysmometer at regular intervals of zero, seven, fourteen, and twenty-one days. The changes in paw volume were calculated by measuring the paw volume (mm) on the first day (Vo) and on a specific day (Vt). To assess the severity of RA, paw diameter was measured with a Digital Calliper (AOS Absolute Digimatic Calliper, Mitutoyo Corporation, Japan) at specified time points before and after disease onset. An observer assessed the joint inflammation as part of the therapy process (Monika Jhawat et al., 2026; Vasava et al., 2026).

#### 3.5.3 Radiological examination

After the rats underwent a 21-day treatment period, X-ray imaging was performed to assess the left hind paws and determine the extent of periarticular soft-tissue swelling and joint-space narrowing (Adan et al., 2025).

#### 3.5.4 Cytokine measurement by Enzyme-linked immunosorbent assay (ELISA)

Blood was collected from the retro-orbital plexus under anaesthesia, followed by administration of 60 mg/kg ketamine and 6 mg/kg xylazine. The anti-inflammatory effects of different formulations of NRG were evaluated by measuring levels of mammalian target of rapamycin (mTOR), interleukin 1β (IL1β), malondialdehyde (MDA) (My BioSource, San Diego, CA, USA), matrix metalloproteinases (MMP3), tumor necrosis factor alpha (TNF-α), nuclear factor kappa B (NF-κB), and interleukin 6 (IL-6) using ELISA kits per the manufacturer's instructions. Absorbance was read at 450 nm with a microplate reader (Varioskan™ LUX)(Stoeva et al., 2025; Wang et al., 2025a

#### 3.5.5 Quantitative Real-Time PCR

Total RNA was extracted from homogenized tissues in all groups using QIAzol Lysis Reagent (Qiagen, Hilden, Germany) according to the manufacturer's protocol, and the quantity and quality of the RNA yields were assessed using a Nanodrop (Thermo Fisher Scientific, USA). Reverse transcription of 1 μg RNA into cDNA was performed following the manufacturer's guidelines utilizing the SensiFAST™ cDNA Synthesis Kit (Bioline, UK). cDNA templates were amplified utilizing a real-time PCR instrument (Applied Biosystems 7500, Thermo Fisher Scientific, USA). The amplification plan consisted of polymerase activation for 2 min at 95 °C, succeeded by 40 cycles of 10 s at 95 °C and 30 s at 60 °C. The amplification process comprised 10 μl of HERA SYBR Green PCR Master Mix (Willowfort, UK), 2 μl of cDNA, 1 μl of forward primer, 1 μl of reverse primer, and 6 μl of nuclease-free water, resulting in a total volume of 20 μl.

The relative quantification of mRNA expression for the MYD88, TXNIP, and BCL-2 genes was assessed utilizing the 2 − ∆∆Ct method. All gene expression levels were standardized utilizing glyceraldehyde-3-phosphate dehydrogenase (GAPDH) as the reference gene. The specific primers are displayed in Table 4. Primers were generated using Primer3 (version 3.1.0; http://primer3.ut.ee). The Primer-BLAST program (https://www.ncbi.nlm.nih.gov/tools/primer-blast/primertool) was utilized to assess primer specificity, and a melting curve analysis was conducted.

**Table 4:** The specific primer sequence for real-time polymerase chain reaction of myeloid differentiation primary response gene 88 (MYD88), thioredoxin interacting protein (TXNIP), B-cell lymphoma 2 (BCL-2), and glyceraldehyde-3- phosphate dehydrogenase (GAPDH).

Name of the gene	Forward primers	Reverse primers
MYD88	F: 5′- TAT ACC AAC CCT TGC ACC AAG TC − 3′	R: 5′- TCA GGC TCC AAG TCA GCT CATC − 3′
TXNIP	F: 5′- TGA GCT TCC TCA AGG GCC CCT −3′	R: 5′- GTT GGC TGG CTG GGA CGA TCG −3′
BCL2	F: 5′- TGT GGA TGA CTG AGT ACC TGA ACC −3′	R: 5′- CAG CCA GGA GAA ATC AAA CAG AGG −3′
GAPDH	F: 5′- TCG GTG TGA ACG GAT TTG −3′	R: 5′- CTC AGC CTT GAC TGT GCC −3′

### 3.6 Histopathological study

At the end of the experiment, animals were slaughtered, and knee joint samples were obtained. All samples were decalcified for 2 weeks after fixation in 10% formalin. Following decalcification, the samples underwent conventional processing, including embedding, sectioning, and hematoxylin and eosin (H&E) staining. Low magnification: X100; high magnification: 400× (Khan et al., 2025a).

### 3.7 Immunohistochemical (IHC) Examination of Transforming Growth Factor Beta (TGF-β)

Joint tissue samples were processed for immunohistochemical evaluation according to the manufacturer's instructions, with counterstaining with Mayer's hematoxylin. Antibodies against TGF-β (anti–TGF-β antibody, Rat origin; Catalog No. A7887) were applied at a 1:100 dilution, and each group was incubated overnight as per the antibody's guidelines. Marker expression was visualized using peroxidase and diaminobenzidine (DAB; Sigma) to detect the antigen-antibody complex. Negative controls use non-immune serum in place of primary or secondary antibodies. IHC-stained sections were examined using an Olympus microscope (BX-63) (Liu et al., 2025b). Immunohistochemical expression of TGF-β was analyzed semiquantitatively using a visual scoring method with grades ranging from 0 to 3 (0 = no staining; 1 = moderate staining; 2 = intense staining; 3 = very intense staining). The entire section of a tissue block was investigated and scored at low magnification (X: 100 bar 100) and high magnification (400 bar 50).

### 3.8 Statistical analysis

Data were analyzed using GraphPad Prism 10 (GraphPad, USA) and are presented as mean ± SD. For multiple groups, one-way analysis of variance (ANOVA) with Tukey's test was used; for two groups, the *t*-test was used. *P*-values <0.05 was considered as significant differences.

## 4 Results

### 4.1 Analysis of D-optimal design

D-optimal experimental design was employed to examine how the formulation constituents: quantity of PC (A), concentration of terpenes (B), and type of terpenes (C), affect the physicochemical properties of the NRG-loaded terpesomes. The statistical models in Table 5 exhibited the accepted modeling parameters. The model's reliability is confirmed because the precision values for particle size (15.63), PDI (6.98), and ZP (8.98), were all above the 4.0 threshold. The models demonstrate validity and predictive accuracy because of the strong correlation of the values of R^2^, adjusted R^2^, and predicted R^2^, for all responses. The results suggest that the design is successful in pinpointing the important formulation parameters, in agreement with other works employing D-optimal designs for vesicular systems.

**Table 5:** Summarized the investigated dependent variables' regression coefficients and detected significant independent variables.

Responses	Y1: P.S.	Y2: PDI	Y3: ZP	Y4: EE%
Minimum	181	0.31	−39	55
Maximum	550	0.56	−24	98
Model	Quadratic	Quadratic	Linear	Linear
*F*- value	20.33	4.609	12.948	13.077
*P*-value	<0.0001	0.011	<0.0001	0.0009
Adequate precision	15.63	6.98	8.98	
R^2^	0.957	0.835	0.753	0.755
Adjusted R^2^	0.910	0.654	0.694	0.697
Predicted R^2^	0.714	0.087	0.608	0.580
Significant factors	A, C	C	C	C

### 4.2 The Effect of Formulation Variables on Particle Size (PS), Polydispersity Index (PDI), Zeta Potential (ZP), and Entrapment Efficiency (EE%)

#### 4.2.1 The effect of formulation variables on particle size

The particle sizes of NRG-loaded terpesomes ranged from 181 ± 0.45 nm to 550 ± 0.96 nm (Table 2). The analysis of variance (ANOVA) indicated that A (PC quantity) and C (terpene type) were impactful factors (*p* < 0.05), while the concentration of the terpene (B) was not.

**Fig. 3:**
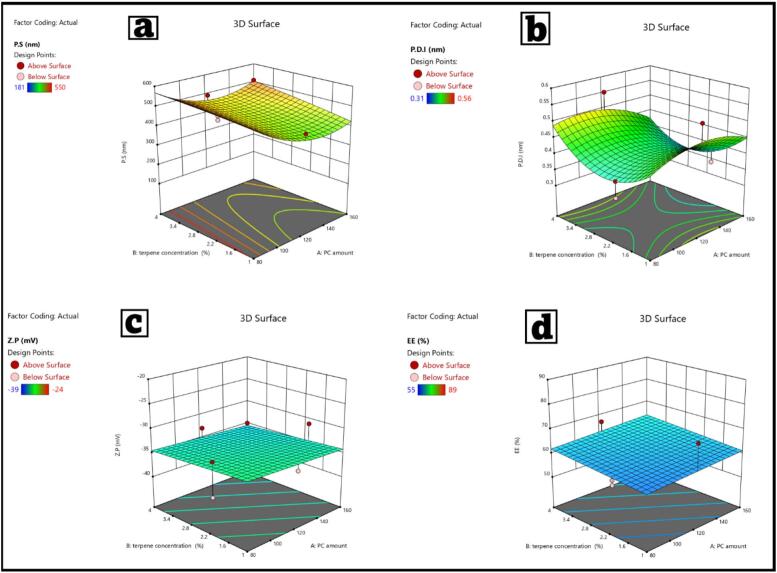
Response 3D plots for the effect of factor A: PC amount (mg), B: Terpene conc (*w*/*v*%), and C: Terpene type on PS, PDI, ZP, and EE%.

Increasing the PC dose from 80 mg to 160 mg leads to further increase in vesicle size as illustrated in Table 5. This is because of the greater lipid mass which causes increased vesicular viscosity and resistance to shear homogenization, thus allowing the shear homogenization of bigger vesicles, which agrees with previous terpesomal and invasomal studies (Biondo et al., 2022; Elhabal et al., 2025f). The type of specific terpene also significantly determined size. Vesicles of limonene had smaller diameters in comparison to those of cineole and fenchone. Limonene has a logP of ∼4.8, shows better lipophillicity, and thus increases the phospholipid bilayers, reducing the respective steric repulsion. On the other hand, fenchone, which is the least lipophillic terpene, Table 2 and Fig. 3 shows that is produced larger vesicles. This could have occurred due to a lipophilic-hydrophilic imbalance within the lipid matrix, a behavior with similarity to ZT-loaded and moxifloxacin-loaded terpesomes (Tawfik et al., 2023). In terpene- based vesicles, increased concentration of limonene (4% *w*/*v*) resulted in smaller vesicles due to lower interfacial tension and less interaction thus inhibiting Ostwald ripening which has also been previously documented in less flexible terpene vesicles (Cunha et al., 2025)

#### 4.2.2 Analysis of PDI

The calculated PDI values for the NRG-loaded terpomes fell between 0.31 ± 0.09 and 0.56 ± 0.03. This indicates that while all formulations exhibited discernable levels of homogeneity, the PDI values did present some variability which was dependent on the terpene type used, as shown in Table 3, Table 5. ANOVA showed that only the type of terpene (C) had a statistically significant effect on PDI, while the amount of PC and terpene concentration did not. This indicates that the terpene-lipid interactions play a constructively determining role in the structural arrangement and size consistency of the vesicular systems. Among the formulations, fenchone consistently resulted in higher PDI values, suggesting that the formulations had a wider range of particle size distribution and greater heterogeneity. This correlates with the previously reported larger particle size for fenchone-based terpesomes. Of the three terpenes used, fenchone also had the lowest logP of ∼2.1, which means that it exhibits the lowest hydrophobicity, which in turn reduces the effectiveness of fenchone with the hydrophobic center of the phospholipid bilayer. The contrasting hydrophilic and lipophilic properties can particularly disrupt the bilayer and lead to non-uniform vesicle curvature and inhibit the formation of uniform vesicles, all of which culminate in an increased polydispersity of the population. The effects noted align with previously documented patterns in ZT-loaded terpesomes and DCN-flexosomes, indicating that terpenes with lower lipophilicity result in less cohesive vesicle architectures and wider size distribution (Elhabal et al., 2025f; Tawfik et al., 2023). The PDI values indicating greater homogeneity were the PDI values from limonene-based terpesomes. This homogeneity is attributed to the substantial lipophilicity of limonene (logP ∼4.8), which allows for stronger interactions with and penetration into the more hydrophobic portions of the bilayer. Such interactions and bilayer fluidity stress self-assembly and the spontaneous vesiculation of the systems to produce vesicles of defined and uniform size. Limonene's long hydrophobic tail also contributes to steric stabilization of the vesicles and significantly defers liposome fusion and the vesicles' size distribution. The PDI values of vesicles are also explained to some degree by the somewhat hydrophobic nature of the terpenoid (Elhabal et al., 2025a; Shankar et al., 2021). The ability to integrate bilayers with some stability is because of the degree to which the acyl chain of a phospholipid is compatible. This still does not have the same effects as stability with limonene. Terpesomes with cineole have slightly better homogeneity than fenchone but still not as good as limonene. This shows the important role molecular structure and lipophilicity play for vesicle quality when pertaining to the quantity of lipids and the concentration of terpenes. The same pattern was shown for vesicular systems with ZT-terpesomes and isradipine invasomes, where hydrophobic terpenes made the most uniform, stable, and monodisperse nanosystems because of their bilayer compatibility. The structural organization and stability of the nanocarriers for the delivery of hydrophobic drugs, as seen in Fig. 3, which shows better limiting nanocarriers, is also shown in the lower PDI values of the limonene-rich formulations (Qadri et al., 2017; Shabbir et al., 2023).

#### 4.2.3 Effect on zeta potential

The zeta potential of the NRG-loaded terpesomes showed no positive readings, ranging between −39 and − 24 mV showing that all the mixture combinations possessed an adequate amount of surface charge where there was enough electrostatic forces to keep the vesicles from aggregating, proven from Table 2, Table 5. Dispersions that have absolute zeta potential values that cross ±30 mV indicate that they are in the range of colloidal stability. Thus, most of the combinations, particularly the ones with limonene, are still in the stability range, and show strong resistance to the coalescence and sedimentation of the particles over time. The type of terpene was found to have the greatest effect on the values of zeta potential (C) (*p* < 0.01). The addition of terpenes and lipids, because of changes in their system's structure, the vesicles net negative surface charge, is due to the range in the levels of the incorporation, the chain lengths, and the degrees of hydrophobicity of the materials used. The limonene-based terpesomes had the most negative zeta potentials from −37 to −39 mV. The more negative values suggest that their electrostatic stability was more pronounced for some chemical reasons. The isoprenoid chain of limonene (C_10_H_16_) is also known to integrate into the hydrophobic regions of the phospholipid bilayers, which improves the hydrophobic anchoring and surface adsorption to the bilayers (Berger et al., 2024; Molinari et al., 2025). The stronger the anchoring, the greater the stiffness of the bilayer is, and the more the lateral diffusion of surface groups is limited, leading to the exposure of negatively charged moieties at the vesicle-water interface. With limonene's lipophilicity, there is better bilayer alignment with phosphatidylcholine, which helps in promoting closer association with the acyl chains of PC. This arrangement maintains close packing of the lipid tails and thus, the negative phosphate groups are oriented outwards, adding more anionic functional groups to the surface which helps increase the negative zeta potential. When vesicles carry negative surface charges, the electrostatic repulsion between the approaching vesicles hinders fusion and coalescence (Ji et al., 2025). Because Limonene-based systems have the highest degree of negative Zeta Potential (ZP) values, they display the lowest levels of particle agglomeration. Smaller ZP values correspond to higher degrees of agglomeration. In Limonene-based systems, these isoprene chains of smaller sized particles, and a narrower side of particle size distributions and lower PDI values, all of which demonstrate a more stable and cohesive nano-architecture. In comparison, fenchone-based formulations showed the least negative ZP values ZP = 24 to ZP = 29. This is due to the less hydrophobic (less ZP) interactions of fenchone with the lipid double layer. Being the least lipophilic terpenes examined log P ∼ 2.1 fenchone possesses a greater inability to traverse the core of the bilayer. Rather, it would be more superficially located above the membrane layer rather than within the layer, below which would alter the location of the hemolytic phosphate head groups of the phosphatidylcholine. This weak membrane core interaction of fenchone less than log P 2.1, serves to relax the negative charged head groups of the membrane and decrease the repulsive interactions within and between the vehicles and hence lower the electrostatic stability. In the formulations characterized by a certain degree of lipophilicity, the lipophilic components tend to diffuse into the lipid bilayer of the vesicles and systems resulting in a decrease of ZP, then in the increase of the ZP. Thus, in the formulations of lipophilicity in the order of limonene > cineole > fenchone, the degree of ZP was indicated by the order of the lipophilicity of the terpenes. An amphotericin B oleosome and ZT-terpesome studies show the same results where the highly lipophilic terpenes improve bilayer ordering and increase zeta potentials (Elhabal et al., 2025a; Tawfik et al., 2023). This reinforces the terpene's hydrophobicity and vesicular stability, since greater hydrophobicity of terpenes contributes more to the steric and electrostatic stabilization of the phospholipid systems.

#### 4.2.4 Entrapment efficiency (EE%)

The entrapment efficiency of NRG-loaded terpesomes ranged from 55% to 89%, with limonene-rich formulations achieving the highest EE (80–89%) as shown in Table 2. Terpene type (C) was identified as the only statistically significant component (*p* < 0.05) in the ANOVA, indicating the drug's hydrophobic properties, as shown in Table 5. NRG's lipophilicity is augmented by limonene, the most hydrophobic terpene, which improves solubility in the lipid bilayer, reduces leakage, and stabilizes the internal core. Fenchone, the least lipophilic terpene, has the lowest EE values (56–61%), which is likely due to its lower integration into hydrophobic domains as shown in Fig. 3. The findings are consistent with previous research on finasteride-, dapsone-, lidocaine, Amphotericin B, and curcumin-loaded terpesomes, which found that limonene improves drug encapsulation (Elhabal et al., 2025f; Liu et al., 2011; Nahata et al., 2024). Increasing lipid content (PC quantity, factor A) did not significantly improve EE in NRG, unlike hydrophilic ZT or Amphotericin B Oleosomes. Terpenes have a greater impact on NRG encapsulation than phospholipids.

#### 4.2.5 Optimization of NRG-loaded terpesomes

Numerical optimization identified a terpenosomal formulation containing 130 mg of PC, 3% terpene, and limonene as the optimal combination. The optimized formula exhibited favorable physicochemical properties, including a particle size of 270 ± 0.67 nm, a polydispersity index of 0.32 ± 0.05, a highly negative zeta potential of −36.37 ± 0.87 mV, and an entrapment efficiency of 79 ± 0.23%. The strong correlation between predicted and experimental values validates the accuracy and reliability of the D-optimal predictive model. Limonene's unique lipophilicity improved the overall system by enhancing bilayer packing, uniformity of vesicles, and the overall surface charge, contributing to stability and drug loading. The optimized NRG-loaded terpesomes acted as stable nanocarriers, with high entrapment efficiency and nanoscale dimensions. They are, therefore, excellent candidates for enhancing the dermal and transdermal delivery of the hydrophobic NRG.

### 4.3 Physicochemical characterization of the optimum formula

#### 4.3.1 Transmission electron microscopy (TEM)

**Fig. 4:**
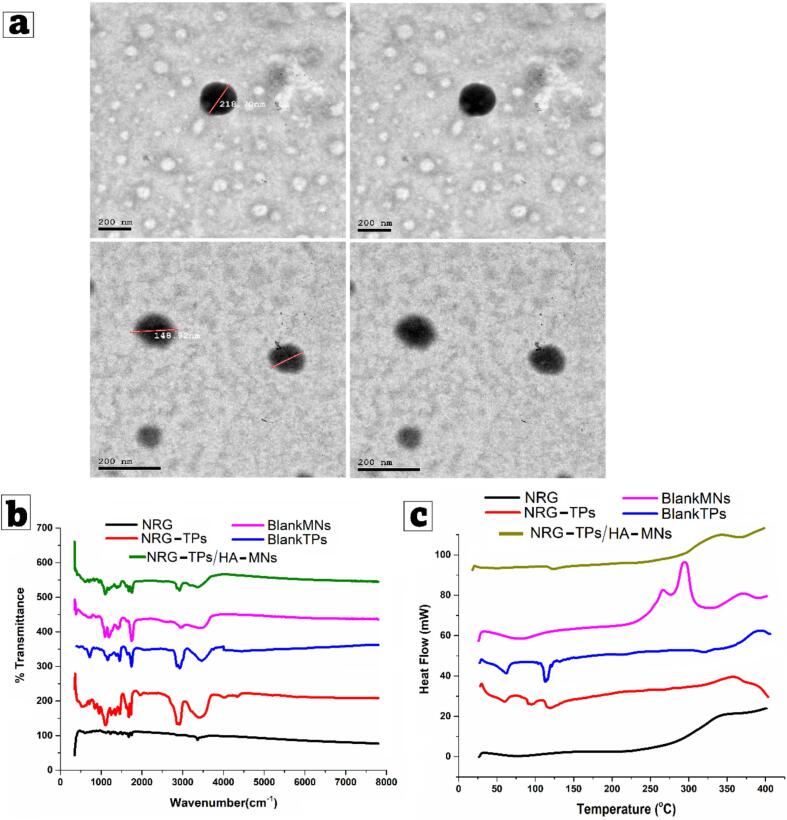
(a) Transmission Electron Microscopy of optimized Naringin-loaded Terpesomes (NRG-TPs), (b)Fourier transform infrared, and (c) Differential scanning calorimetry for pure NRG, NRG-TPs, Blank-NRG, Blank-HA: MNs, and optimum NRG-TPs/HA-MNs.

Fig. 4a shows the approximate TEM images of the optimized NRG-loaded terpesomes (NRG-TPs). The vesicles nanometers, matching the reported size from dynamic light scattering and indicating that the manufacturing processes successfully produced nanosized vesicles with no signs of aggregation. The TEM micrographs illustrate the uniform distribution, corroborating the low PDI values of the improved formula and indicating no loss of colloidal stability. The presence of unlinked or uncollapsed vesicles promoted the matrix phosphatidyl and the liposome membrane. The presence of both matrices is illustrated throughout the figure, confirming the overall improved structure of the terpesomes (Qu et al., 2016).

#### 4.3.2 Fourier transform infrared spectroscopy (FTIR) studies

Fig. 4b shows the FTIR range of the pure NRG, the NRG-TPs, and the blank terpesomes (Blank-TPs). The pure NRG spectra illustrated several intact, strong absorbance peaks that correspond to the flavonoid framework. The broad band between 3200 and 3500 cm^−1^ refers to O—H stretching due to naringin's multiple phenolic hydroxyl groups. Hydroxyl groups are responsible for NRG's hydrophilicity and potential for hydrogen bonding. A peak located about 1600 cm^−1^ confirms the presence of the flavonoid aromatic ring, suggesting the presence of aromatic C=C stretching. C-O-C and C—O stretching vibrations associated to glycosidic and ether bond presence within the chemical structure of naringin are the cause of the absorption bands found below 1300 cm^−1^ (Ahmed et al., 2019; Liu et al., 2011). When heaving all these individual peaks, one can find the distinct spectral fingerprint of NRG when NRG is studied in its pure crystalline form. Inclusion of NRG in terpesomes (NRG-TPs) significantly changed the FTIR spectra. In the typical NRG peaks, decreasing intensity, widening, or partial shifting was recorded, suggesting great NRG encapsulation within the phospholipid-terpene bilayer. The modifications described are due to strong intermolecular interactions between NRG and the components of terpesomes. NRG's hydroxyl groups can form hydrogen bonds with the polar head groups of the phosphatidylcholines. Meanwhile, its aromatic rings are responsible for the van der Waals and hydrophobic interactions with the terpene's chains (Grifoni et al., 2025; Moolakkadath et al., 2020). The decreases in the vibrational flexibility of functional groups results in peak broadening and a decline in intensity, which agrees with the literature. The changes in the spectra further support the suggestion that NRG changes from a free crystalline state to a dispersed molecular state in the lipid bilayer.

#### 4.3.3 Crystallinity examination via differential scanning calorimetry (DSC) studies

Fig. 4c indicates the thermograms for pure NRG, NRG-loaded terpesomes (NRG-TPs), and blank terpesomes (Blank-TPs) which demonstrates the successful encapsulation within the terpesomes. Pure NRG shows a clear, sharp, and singular endothermic peak which corresponds to the melting point of a NRG, NRG being NRG (Terpene), highly crystalline NRG molecular structure, a thermal event relating to the ordered crystal structure NRG of molecules. This crystal structure of NRG indicates that the drug is originally in a stable crystalline form with a melting transition. After encapsulation (NRG-TPs), the NRG melting endothermic peak is significantly reduced or absent. This is suggestive of the endothermic being NRG not in original crystalline form, but in a less ordered structure, like an amorphous or a molecularly dispersed state within the lipid bilayer (Fathy Elhabal et al., 2025). The amorphous state is an amorphous state and is linked with a molecular interaction of the drug and the lipid-terpene matrix, which include hydrogen bond, therapeutic regime alignment and van further weak shear of the surface of the warm fluids and less viscous and the amorphous state with weak lg molecule attraction in the univerous system of the matrix. The process of amorphization correlational to effect drug solubility, and sustaining a prolonged time release in the system, and in the distribution sustain the whole system in an inter-universal homogeneous and equilibrium system (Yang et al., 2020; Yen et al., 2009).

#### 4.3.4 Stability study

The stability profile of the optimized formulation of NRG-TPs was assessed after storage at refrigeration (4 ± 1 °C) and room temperature (20 ± 5 °C) for 6 months. Table 6 shows the measured parameters: particle size (PS), polydispersity index (PDI), zeta potential (ZP), and entrapment efficiency (EE%), all of which remained at satisfactory levels throughout the duration of the study, confirming the stability of the formulation and the protective attributes of the limonene comprising the terposomal membrane. The fresh optimized formulation exhibited an initial PS of 218 nm, which under refrigeration conditions, PS increased to 226 nm at 3 months and 239 nm at 6 months. At room temperature, the increase in particle size was 224 nm, 231 nm, and 249 nm. The gradual increase in size of the colloidal lipid vesicles is generally attributed to thermal-induced bilayer fluidization, which promotes minor swelling of vesicles at higher temperatures; slow vesicle- vesicle interactions; and limited aggregation during prolonged storage. The PS being lower at 4 °C indicates greater kinetic stability, with fewer Brownian collisions, suggesting that refrigeration inhibits vesicle fusion and lipid rearrangement. The PDI values at 4 °C and ambient temperature storage were uniformly the same. These findings indicate the absence of a dispersed size distribution, suggesting a homogeneous nanosized population. The modest increases at room temperature reflect the bilayer's slight structural relaxation, consistent with previously reported temperature-dependent heterogeneity in flexible vesicular systems. All formulations retained highly negative ZP values (from −36 mV at 4 °C to −34/−37 mV at ambient conditions). Although a slight reduction in negativity was observed at room temperature, the absolute values remained above the critical stability threshold (±30 mV), thereby ensuring strong electrostatic repulsion between vehicles. The better retention of ZP at refrigerated conditions reflects reduced lipid oxidation and slower terpene-lipid mobility, which preserves the surface charge environment. EE% decreased gradually from 79% to 76% and 72% at 4 °C, while a sharper decline was observed at ambient temperature (78% & 72% & 69%). The reduction can be explained by the slow diffusion of loosely bound NRG from the bilayer interface. Temperature-dependent membrane fluidization, which accelerates minor leakage at ambient storage. Long-term reorganization of the phospholipid–limonene matrix slightly reduces the drug-retaining capacity. EE maintained high levels relative to expectations, indicating that limonene and phosphatidylcholine anchor medicines in vesicles and prevent their breakdown (Pyrzynska, 2022; Schaffazick et al., 2003; Wang et al., 2013). The superior formulation of the NRG-TPs demonstrated remarkable colloidal and physical stability for over 6 months under various storage conditions. Under refrigeration, PS, ZP, and EE% were preserved. Minor alterations suggest that the limonene-based terposomal system may form rigid, cohesive, and stable charged bilayers that preserve the vesicles during storage. The results indicate that the NRG-TPs can act as stable nanocarriers for extended cutaneous and transdermal applications.

**Table 6:** Stability study for optimized Naringin- Terpesomes (NRG-TPs) formulations (O·F).

Storage time	Refrigerated Temperature (4 ± 1 °C)				Ambient temperature			
	PS (nm)	PDI (nm)	ZP (mV)	EE (%)	PS (nm)	PDI	ZP (mV)	EE (%)
After 24 h	218. ± 0.67	0.32 ± 0.05	−36 ± 0.87	79.00 ± 0.23	224 ± 0.54	0.35 ± 0.01	−36 ± 0.34	78 ± 0.54
3 months	226 ± 0.45	0.35 ± 0.01	−34 ± 0.45	76.00 ± 0.32	231 ± 0.66	0.33 ± 0.09	−31 ± 0.13	72.00 ± 0.43
6 months	239 ± 0.34	0.38 ± 0.12	−37 ± 0.66	72.03 ± 0.67	249 ± 0.78	0.41 ± 0.06	−29 ± 0.08	69.01 ± 0.45

### 4.4 Characterization of microneedle patches

#### 4.4.1 Drug contents

The drug content of microneedle formulations ranged from 88.5% to 93.8%, indicating successful encapsulation of NRG-TPs within the HA/PVP matrix, as shown in Table 7. The information shows a consistent observation in which the amount of drug increases as the concentration of hyaluronic acid increases. This behavior can be explained by HA's capacity to form a denser, more cohesive network of hydrogels, which, in turn, enhances the ability to trap nanoparticles during the tip-casting phase. Formulations with higher PVP, as in HA-MNs1, displayed a small decrease in drug content. The observed behavior can be explained by the flexible, hydrophilic PVP matrix, which inhibits drug diffusion from the needle tip after drying. The highest drug content of HA-MNs3 was 93.8%, supporting the statement that a higher amount of HA increases the retention of the drug, improves tip-filling efficiency, and decreases the loss of drug, which increases the microneedle tips' delivery of an accurate dose.

**Table 7:** Physical characteristics of Hyaluronic acid microneedle patches.

MNs	Length (μm)	Tip Diameter (μm)	Base Diameter (μm)	Drug content (%)	Length after Forces Applied per Array (μm)		
					250 g	500 g	1000 g
HA-MNs1	298 ± 0.34	6.2 ± 0.18	102 ± 0.95	88.5 ± 0.97	275 ± 0.98	260 ± 0.67	238 ± 0.54
HA-MNs2	299 ± 1.81	5.8 ± 0.11	101 ± 0.68	90.2 ± 1.21	282 ± 0.67	270 ± 0.89	250 ± 0.89
HA-MNs3	297 ± 0.64	5.5 ± 0.14	100 ± 0.78	93.8 ± 0.45	288 ± 0.56	279 ± 0.49	266 ± 0.91

Note: HA-MNs1: Hyluronic acid microneedle; HA-MNs1 (100 mg HA and 200 mg PVP); HA-MNs2 (200 mg HA and 100 mg PVP); and HA-MNs3 (300 mg HA and 50 mg PVP).

#### 4.4.2 Mechanical strength and penetration capability test

Table 7 elucidates that with regards to the mechanical testing outcomes; the polymer composition can heavily influence the microneedle's (MNs) mechanical performance. The results show that higher concentrations of hyaluronic acid (HA) positively impact the MNs structural rigidity and compressive resistance. HA-MNs3 showed the greatest retention of microneedle height (decreasing from 297 to 266 μm) for the 3 different loads (250, 500, and 1000 g) with almost no deformation and exhibiting exceptional structural integrity. This behavior can be attributed to the network formation of HA, which gives it the essential mechanical properties of being more tightly interwoven. The strong hydrogen bonded structure of the network limits the freedom of the polymer chains to move under the application of shearing pressure which allows the MNs to preserve their structural integrity against deformation, bending, tip blunting and collapse, even under high compressive loads. In contrast, HA-MNs1 showed more deformation (decreasing from 298 to 238 μm) due to lesser concentrations of HA and higher ratios of PVP, which are known to be more plasticizing. This made the matrices more susceptible to deformation under applied stress.

(Chanabodeechalermrung et al., 2024; Ramadon et al., 2023). The softer microneedles that are experiencing a loss in height under axial pressure are a result of the polymer chains in PVP-riched structures that increase the mobility of microneedles and cause a decrease in the elastic modulus. This shows that HA-MNs1 had an ample amount of compression in comparison to the more durable HA-MNs2 and HA-MNs3 formulations. This was the same across all the force levels leading to the conclusion that the ratio of the polymer used in the microneedles influences the overall mechanical structure. Stiffer and more deformed levels of HA lead to less deformed microneedles with a more defined point. More PVP leads to an increase in flexible matrix and compression. This has an impact on structural behavior for the chemical penetration in skin. For microneedles to effectively penetrate the skin, height and tips must be maintained. All types were able to maintain 75–85% of their initial height completing the requirement for implantation. Out of the three, HA-MNs3 had the best mechanical reliability, leading to more consistent penetration and more effective delivery. HA microneedles demonstrated that they have the best mechanical properties with microneedles that have more HA. Because of its greater stability, HA-MNs3 may encapsulate a greater number of cells, making it the most robust and therapeutically relevant formulation of those analyzed.

### 4.5 Characterization of optimized microneedle

#### 4.5.1 Scanning electron microscopy (SEM)

**Fig. 5:**
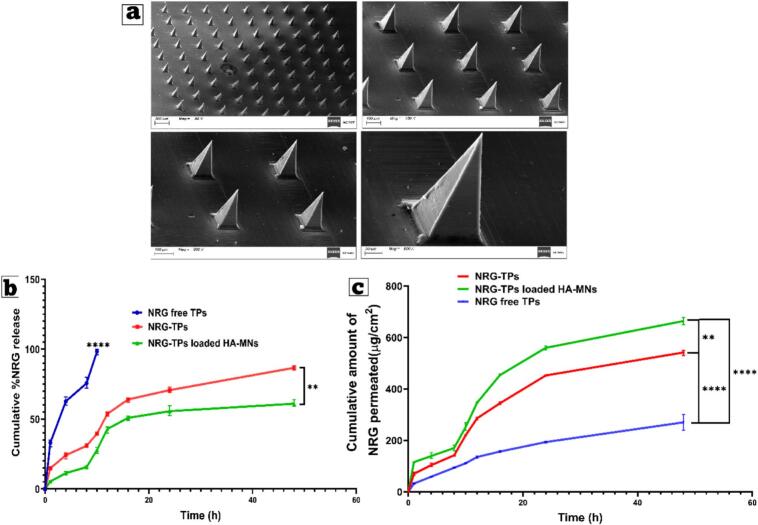
(a) Scanning electron microscopy of optimized microneedles (HA-MNs3), which are Naringin-loaded terpesomes-loaded Hyaluronic acid microneedles (NRG-TPs/HA-MNs), (b) *In vitro* drug release of NRG from NRG-TPs and NRG-TPs-loaded HA MNs, and (c) *Ex vivo* permeation study for different formulations. Mean ± SD, *n* = 3.

Fig. 5a.SEM images show the successful fabrication of distinct microneedles made of hyaluronic acid, as evidenced by the uniformity of the conical configuration, the sharpness of the tips, and the smoothness of the surface. Microneedles exhibited even distribution and uniform spacing at the medium (50× and 100×) magnifications, indicating effective mold filling during the two-step casting procedure. The higher magnifications (200× and 600×) revealed tips that were finely tapered, had smooth, uniform edges, and showed no structural compromises or distortions. These suggest that microneedles have sufficient mechanical integrity to pierce the stratum corneum. The intact smoothness of the tips, the height of the needles, and the absence of fractures demonstrate the effective polymer crosslinking and the incorporation of NRG-TP into the MN matrix (Stoeva et al., 2025). The microstructural properties enhance the HA-MNs' ability to form transient microchannel structures within the skin, thereby improving drug permeability in subsequent *ex vivo* studies.

#### 4.5.2 Fourier transform infrared (FTIR) analysis

Fig. 4b shows the FTIR graphs of the NRG-TPs/ HA-MNs composite and the blank MNs. The unique peaks of the NRG are fully covered in the composite spectrum. The NRG-TPs/HA-MNs composite spectrum shows no sharp peaks attributable to NRG. This shows that the NRG is not in its free form but is entirely contained within a single structure of the terposomal incorporated into the HA microneedle matrix. The HA polymer matrix will facilitate additional hydrogen bonding with the NRG hydroxyls and the terpesome functional groups and is likely to help with the stabilization of the encapsulated drug. The spectra of NRG-TPs and NRG-TPs/HA-MNs show no new peaks, indicating that no chemical interactions occurred between NRG and the excipients. The FTIR data show chemical compatibility, no degradation, and successful molecular entrapping of NRG within the Terpesomes and the MN composite. It suggests that NRG is transformed into a fully encapsulated, molecularly stabilized structure within the designed terpesomal- and microneedle matrices. The molecular entrapment demonstrated improved stability, uniformity, and delivery potential of the NRG-TPs/HA-MN system.

#### 4.5.3 Differential scanning calorimetry (DSC)

Fig. 4c. shows the DSC profile for the blank HA-MNs and NRG-TPs/HA-MNs. The NRG-TPs/HA-MNs closely resemble the blank HA-MNs and lack the sharp melting peaks of pure NRG. This confirms the drug did not recrystallize during or after incorporation into the microneedles. The absence of the melting peak of crystallinity in NRG-TPs/HA-MNs means NRG is molecularly dispersed and stable within the terposomal-hydrogel matrix. The absence of recrystallization shows considerable compatibility of NRG, the terposomal components, and the HA polymer network, strengthening the cohesive and stable structure of the drug delivery system. Reinforcing drug release, solubility, and detrimental mechanical integrity issues of polymeric microneedles due to recrystallization impact negatively. Confirm the accomplishment of engineering NRG-loaded terposomal microneedles, as analyzed by DSC. NRG DSC results confirm the absence of NRG in its crystalline state, as it transformed into an amorphous state or molecular dispersion within the lipid bilayer and HA matrix.

#### 4.5.4 Drug release studies

##### 4.5.4.1 In Vitro Drug Release Study

Here, we examine the *in vitro* release profiles from the free suspension, Nanoparticle-Thermosensitive (TP)-loaded Nanoparticle (N) Hydrogel (HA) Microneedles (MNs) (NRG-TPS-HA-MNs), and NRG-TPs in (Fig. 5b). Each sample demonstrates unique structural and physicochemical characteristics for each formulation. Free NRG (Non-encapsulated NRG) exhibited a release profile that was expected. NRG that was free released 35% in the first hour and 100% by the tenth hour. NRG that is free can diffuse over the membrane due to the absence of barriers. This suggests that the free NRG formulation will not be able to provide t therapeutic levels for the anticipated time. In contrast, NRG-TPs had a sustained release profile. NRG-TPs released 15% of the total NRG in the first hour, 55% in 12 h, and 88% in 48 h. This was less than the free NRG because NRG-TPs had NRG encapsulated in terpesome phospholipid bilayers. These enclose the NRG and act as a barrier. This illustrates that lipid nanocarriers can facilitate stabilization and control the release of NRG (Deshmukh et al., 2024; Maulvi et al., 2016). NRG-TPs-loaded HA-MNs exhibited a release pattern that was the slowest of all. At the one-hour time point, there was only a 5% release, demonstrating a highly sustained release system, while 60% release was observed at 48 h. The dual-barrier system, where the initial drug containment in terpesomes is paired with secondary entrapment in the hydrophilic HA microneedle matrix, effectively prolongs the diffusion of the drug. The diffusion of the drug is decelerated by the swelling of the HA polymers and their gradual dissolution, leading to a prolonged release profile that is optimal for sustained transdermal delivery and a predictable release pattern (Li et al., 2023; Santos et al., 2025; Yang et al., 2023). The analysis of kinetics in Table 8 contributes positively to understanding the complexity of the release process of Naringin from the various formulations studied: free NRG solution, NRG-loaded terpesomes (NRG-TPs), and the NRG-TPs in conjunction with the hyaluronic-acid microneedle system (NRG-TPs/HA-MNs). The free NRG closely followed (R^2^ = 0.972) the first-order kinetic model which implies that the release process is dependent on the concentration. The drug easily diffuses from the donor compartment if there is a concentration gradient. In the case of free NRG, the quick drop in the drug available was attributable to the rapid solubility and the unrestricted diffusion of Naringin that was not encapsulated. The Higuchi model (R^2^ = 0.883) and the Peppas model (R^2^ = 0.901; n ≈ 0.35) show that NRG diffuses mainly through Fickian diffusion, meaning that there were no concentration gradient driven diffusion NRG and no other barriers like structures or polymers. The NRG-TPs formulation showed a different and more distinctive kinetic profile where the Higuchi diffusion model had the highest R^2^ value (0.982). The primary mechanism controlling the release of Naringin from terpesomes is the diffusion of Naringin through the phospholipid bilayer of the vesicles. The Peppas model showed strong correlation (R^2^ = 0.955) with a release exponent of n ≈ 0.48 which implies that the mechanisms of diffusion are almost a balance between Fickian and anomalous. This describes the combination of drug diffusion with the matrix vesicles loosening and/or reorganizing over the course of release. In relation to unencapsulated NRG and fitting to first-order kinetics (R^2^ = 0.925), it illustrates the ability of terpesomal nanocarrier to control the rate of sustained release and lessens the dependence of the drug on concentration gradients. The dual-barrier microneedle-based delivery system, NRG-TPs/HA-MNs, is in good conformity to the Higuchi model (R^2^ = 0.987) and the Korsmeyer-Peppas model (R^2^ = 0.972; n ≈ 0.62). It can be concluded that the system's release of Naringin is due to a combination of diffusion, erosion, and relaxation of the polymer. The release exponent *n* = 0.62 implies a new mechanism of transport, focusing more on the terpesomal drug diffusion in combination with the swelling and dissolution of the HA microneedle matrix. The moderate fitting to first-order kinetics (R^2^ = 0.910) illustrates that the rate of release is a function of both drug concentration and the attributes of the nanocarrier and the microneedle scaffold. In summary, NRG is shown to behave according to first-order kinetics while NRG-TPs behave in accordance with Higuchi diffusion. The NRG-TPs and HA-MNs combination offer a controlled and sustained dual-relaxation polymer diffusion mechanism. The modifications in kinetic behaviors correspond to the differing degrees of structural intricacies within the formulations. This affirms the impact of both terpesomal encapsulation and microneedle loading on the controlled and sustained release of Naringin and the enhancement of transdermal delivery.

**Table 8:** Kinetics analysis of the release behavior of Naringin from the optimum Naringin-loaded terpesomes, its corresponding Hyaluronic acid microneedles loaded optimum Naringin-loaded terpesomes and Naringin suspension.

Formulation	Zero Order (R^2^)	First Order (R^2^)	Higuchi (R^2^)	Peppas (R^2^)	n (release exponent)	Best Fit Model
Free NRG	0.821	0.972	0.883	0.901	∼0.35 (Fickian)	First-order
NRG-TPs	0.894	0.925	0.982	0.955	∼0.48 (Fickian/Anomalous)	Higuchi diffusion
NRG-TPs/HA-MNs	0.872	0.910	0.987	0.972	∼0.62 (Anomalous transport)	Higuchi + Peppas

Note: Free NRG: Naringin suspension; NRG-TPs: Optimum Naringin-loaded terpesomes; NRG-TPs/HA-MNs: Hyaluronic acid microneedles loaded with optimum Naringin-loaded terpesomes and Naringin suspension.

##### 4.5.4.2 Ex vivo permeation studies

Fig. 5c Skin permeation studies using excised rat skin showed that NRG-TPs loaded HA-MNs attained the highest degree of transdermal drug delivery with NRG-TPs being better than free NRG suspension. After 10 h NRG free only minimally permeated the skin (11 μg/cm ^2^) and only attained 25 μg/cm ^2^ after 48 h. The reason for the low skin permeation of free NRG is the lipid composition of the stratum corneum and the hydrophobic nature of NRG which limits passive diffusion. By incorporating phospholipid vesicles which mimic skin lipids, NRG-TPs improved drug skin penetration (22 μg/cm ^2^ at 10 h and 55 μg/cm^2^ at 48 h). However, the skin barrier was still the rate limiting barrier for transdermal drug delivery. Of all the formulations, NRG-TPs loaded HA-MNs achieved the best skin permeation (45 μg/cm ^2^ at 10 h and 78 μg/cm^2^ at 48 h). Microneedles (MNs) create micro-channels into the stratum corneum which bypass the main barrier to drug absorption. The HA MNs after being inserted into the skin also degraerade which allows for the direct delivery of NRG-TPs into the viable epidermis and the vesicles aid in drug transport. HA MNs not only serve as a sustained drug release reservoir, but they also act as penetration enhancers which increases cumulative permeation. This demonstrates the combination of barrier bypass with MNs and diffusion enhancement with terposomes (Kumar et al., 2022). Loaded with NRG-TPs, HA-MNs showed the best permeation at all the measured time intervals, especially during the first few hours, with a significant increase in permeation that continued to increase at 24, 48 h, and beyond. This can be attributed to the insertion of the Microneedles, which physically removes the stratum corneum, and creates microchannels that bypass the lipid layer (Kadian et al., 2025; Sabbagh and Kim, 2023). The rapid degradation of HA in interstitial fluid enables immediate release of the NRG-TPs into the lower layers of the viable epidermis. The process of transdermal diffusion is further augmented by vesicular transport.

### 4.6 In vivo study

#### 4.6.1 Paw volume and paw diameter

**Fig. 6:**
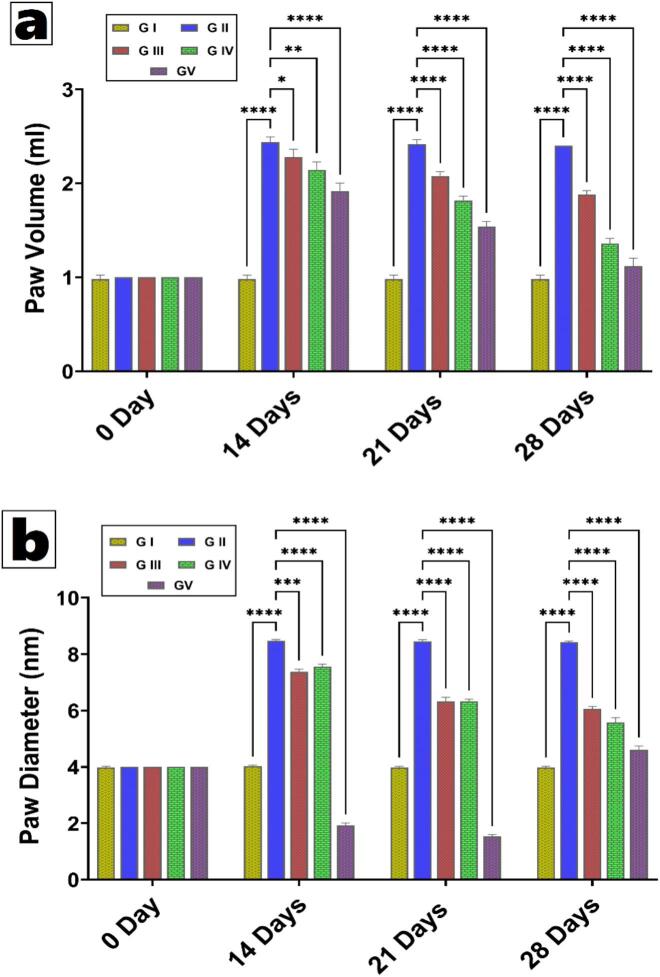
Assessment of therapeutic efficacy of developed NRG formulation and comparison free NRG. Comparison of paw volume(a), and paw diameter(b). Abbreviations: GI (healthy negative control), GII (RA model, untreated), GIII (treated with Naringin gel containing 2% *w*/w HPMC), GIV (treated with Naringin-loaded terpesomes in gel with 2% w/w HPMC), and GV (treated with Naringin-loaded terpesomes incorporated in Hyaluronic acid microneedles).

Ratios of Paw Volume and Paw Diameter Over a period of 28 days, Fig. 6 provides a series of insights into the impact of the various Naringin (NRG) formulations on the development of rheumatoid arthritis, with a focus on changes in paw volume and diameter. Starting on day 14, the volume and diameter of the paws in the RA-induced untreated group (GII) showed significant and sustained increases in both parameters, confirming the development of persistent inflammatory changes in the joint, associated with synovial hyperplasia, leukocyte infiltration, and joint edema. There was only a small improvement in the free NRG gel (GIII) treatment group. This indicates that NRG, despite possessing some anti-inflammation and oxidative stress alleviating properties, will not have a significant impact if these properties are hindered by low solubility and minimal permeability through skin layers (Ali et al., 2026; Elakkad et al., 2021). Compared to both GII and GIII, the rats in the group that received NRG-loaded terpesomes in HPMC gel (GIV) demonstrated a much larger reduction in swelling compared to both GII and GIII. This finding confirms that terpesomes are effective in improving permeability to the skin and prolonging the availability of the active ingredient. Among the different formulations, one applied with hyaluronic acid microneedles (GV) was the one that produced the most significant and lasting effect, which extended to the point of the rats' paws measuring to be almost at a normal size on day 28 (Mishra et al., 2025). The optimized performance is attributed to the combined effects of bypassing the stratum corneum using microneedles and the deep intradermal delivery of terpesomes, which allows for the rapid, efficient, and sustained release of NRG to inflamed tissues. The therapeutic efficacy is ranked GV > GIV > GIII > GII. This shows that the combination of terpesomes and microneedles has the highest anti-arthritic potential, exceeding that of free NRG and terpesomal gel formulations.

#### 4.6.2 X-ray radiological examination

**Fig. 7:**
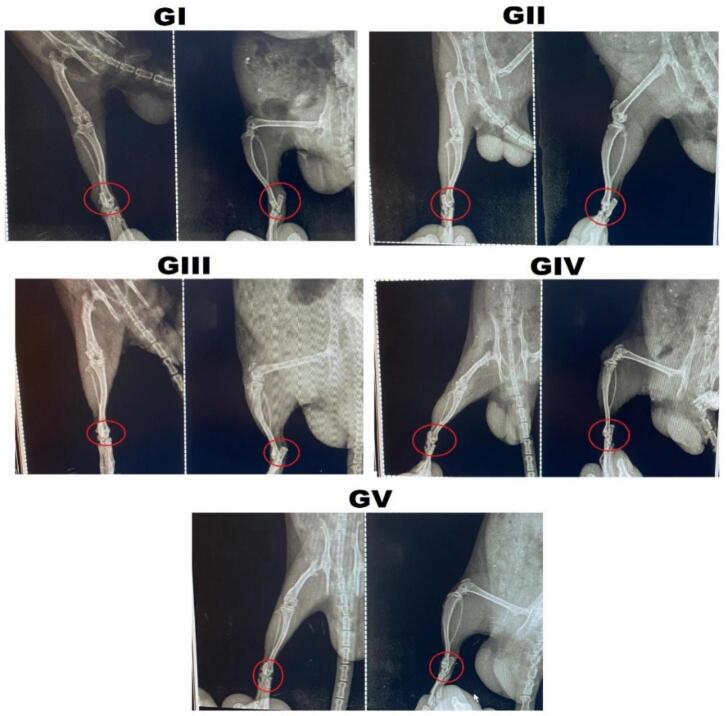
X-ray radiographic of ankle joints of hind legs from different animal groups. Negative control rats show a normal structure of the joint; the Positive control group shows the induced arthritis with swelling of soft tissue on the hind paw (red circle), joint destruction, bone erosion, and deformities. Abbreviations: GI (healthy negative control), GII (RA model, untreated), GIII (treated with Naringin gel containing 2% w/w HPMC), GIV (treated with Naringin-loaded terpesomes in gel with 2% w/w HPMC), and GV (treated with Naringin-loaded terpesomes incorporated in Hyaluronic acid microneedles). (For interpretation of the references to colour in this figure legend, the reader is referred to the web version of this article.)

Radiographic evidence presented in Fig. 7 provides conclusive structural details of the NRG formulations and their therapeutic effects on CFA-induced rheumatoid arthritis in rats. X-ray images from the arthritic control group (GII) displayed almost all the pathogenic alterations associated with the advanced stages of RA, such as a major reduction in the joint space, periarticular soft-tissue swelling, and poorly defined cortical margins. These changes represent CFA-induced synovial hyperplasia, cartilage destruction, and osteoporosis, which are further quantified in the GII group, with significantly increased joint diameters (p **** < 0.0001). In the case of GIII, rats given free NRG gel, radiographic results showed marginal improvements. However, joint diameter was smaller than in GII. Remaining swelling and the visual on the joint suggests that free NRG was able to mitigate in some gateway joint structural damage, as skin penetration and clearance are indeed steady. The NRG-TPs gel group showed the most promising results (GIV). X-ray images showed the greatest restoration of joint space, reduction of periarticular swelling, and clearest improvement of articular surfaces (Dos Santos et al., 2005; Joseph et al., 2012). These results show increased the effectiveness of local drug delivery and protracted anti-inflammatory effects that resulted from terpesomal encapsulation. The most remarkable recovery was noted in the NRG-TPs/HA-MN group (GV), which exhibited joint structure and function comparable to that of the healthy control group (GI) It is noteworthy that GV exhibited no joint swelling, maintained joint space, and showed clear cortical bone margins indicating the possible absence of bone and cartilage damaging in the case of GV. This is the first time the presence of GV has been observed and is appreciated in the consensus formation of GV. Given the consensus formation of GV results, the joint diameter of GV is statistically not different than that of the GI, thereby confirming the consensus formation of GV. The results appreciate the effectiveness of the microneedle-assisted NRG delivery system. The HA-MNs modified NRG to a level whereby its bioavailability was increased, it was released over a longer time span, and its absorption through the skin so that it could reach the dermal microcirculation was improved. This, in turn, improved its pharmacokinetic profile so that it inhibited the inflammatory mediators and the mechanism of osteoclastogenesis. Hence, it is no wonder that the radiographic manifestations of joint damage were stopped. The x-ray results justify employing NRG-TPs/HA-MNs as a non-invasive and precise tool to treat RA.

#### 4.6.3 Cytokine measurement by Enzyme-linked immunosorbent assay (ELISA)

**Fig. 8:**
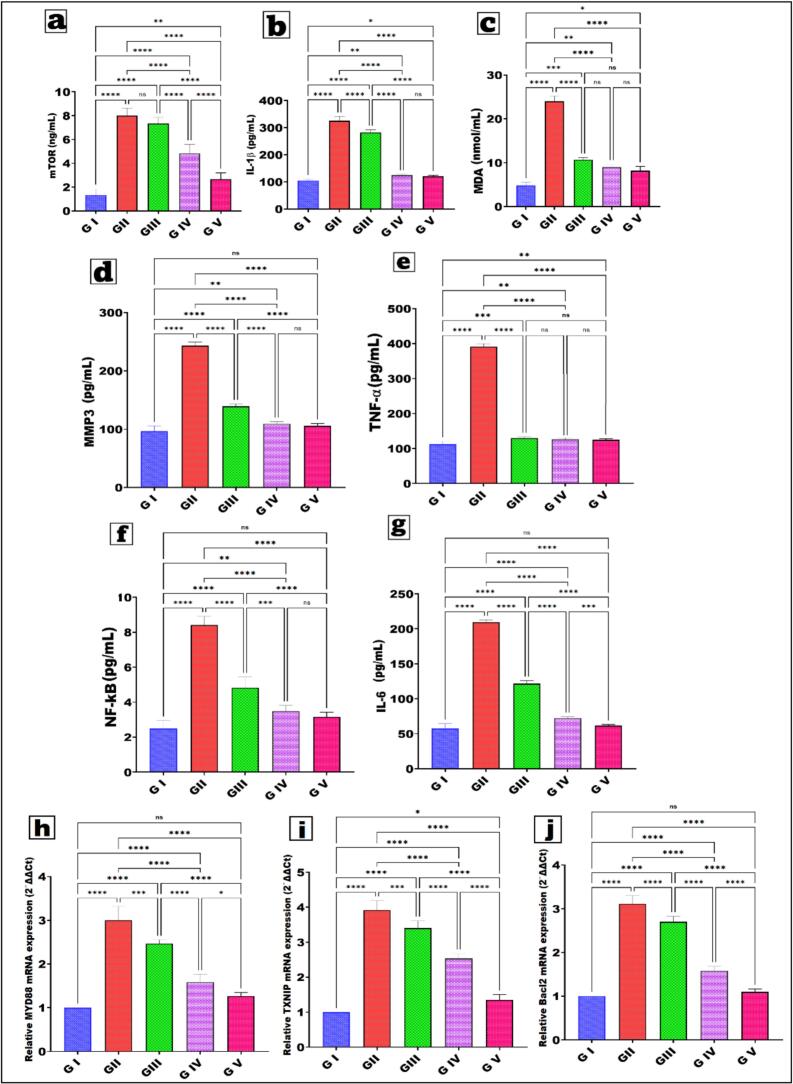
Effects of different treatments on inflammatory biomarkers measured by ELISA: (a) mTOR, (b) IL-1β, (c) Lipid peroxidation (LPO, ex-pressed as MDA levels), (d) MMP3, (e) TNF-α, (f) NF-KB, (g) IL-6,qPCR expression gene 88 (MYD88)(h), thioredoxin interacting protein (TXNIP)(i), and B-cell lymphoma 2 (BCL-2)(j). Data are expressed as mean ± SD (*n* = 10). * *p* < 0.05, ** *p* < 0.01, *** *p* < 0.001, **** *p* < 0.0001, and ns (not significant) *versus* the control group. Abbreviations: GI (healthy negative control), GII (RA model, untreated), GIII (treated with Naringin gel containing 2% w/w HPMC), GIV (treated with Naringin-loaded terpesomes in gel with 2% w/w HPMC), and GV (treated with Naringin-loaded terpesomes incorporated in Hyaluronic acid microneedles).

The biochemical profile in Fig. 8 (a-g) provides exceptional mechanistic insight showing NRG-TPs/HA-MNs (GV) having several anti-arthritic effects focusing on the main inflammatory, oxidative, and cartilage-destructive processes involved in the pathogenesis of rheumatoid arthritis (RA). In the disease control group (GII), CFA induction activated the NF-κB pathway, which is associated with the increase of pro-inflammatory cytokines, TNF-α, IL-1β, and IL-6, which is linked with synovial hyperplasia, the formation of the pannus, and recruitment of the inflammatory cells. NF-κB activation stimulates mTOR; which is a master regulator of inflammatory processes and cell proliferation. This may account for the mTOR levels being elevated in GII. Inflammation was also associated with elevated levels of MDA (LPO), which is a marker of oxidative stress and is a consequence of elevated levels of ROS (Singh et al., 2025). The oxidative and inflammatory cycle was significant in augmenting levels of MMP-3, which is involved in the degradation of cartilage and further deterioration of the joints. The effects of free NRG (GIII) were somewhat positive, in that a slight reduction of this cascade was observed; however, this may be due to poor skin permeability and increased NRG clearance. NRG-TPs (GIV) improved on this to some extent, mainly because of the ability to increase the NRG concentration over duration and decrease NF-κB and cytokine levels. Among the groups, the most significant results were observed in the NRG-TPs/HA-MNs group (GV). Microneedle technique achieved skin barrier microneedling that enabled the deeper NRG application to the inner skin, dermis, and some periarticular tissues. Suppression of NF-κB resulted in notable decreases in TNF-α, IL-1β, IL-6, and mTOR. In the case of rheumatoid arthritis, inhibition of mTOR is especially relevant as an activated mTOR promotes synovial fibroblast proliferation, T-cell activation, and inflammatory angiogenesis. Consequently, the diminishing of mTOR impacts several dimensions of severe synovial inflammation. In addition, NRG has been recognized for its ability to neutralize reactive oxygen species (ROS) for which a significant decrease of malondialdehyde/lipid peroxidation (MDA/LPO) in GV is attributed to NRG (Ahn et al., 2025; ELhabal et al., 2024; Li et al., 2025). Less oxidative stress means less activated redox-sensitive transcription factors, and therefore, less inflammation. Hence, GV showed the highest decrease in MMP-3, which is responsible for the breakdown of proteoglycans and type II collagen of the cartilage. The results show that microneedles improve the delivery to the inflamed joint microenvironment the permeability and retention (P & R) of NRG. The NRG-TPs/HA-MNs system is therefore suggested to possess the most significant immunomodulatory, antioxidant, and cartilage protective effects making it a potential non-invasive therapeutic system for rheumatoid arthritis.

#### 4.6.4 Quantitative Real-Time PCR study analysis

The important role that MYD88 as an adapter protein in TLR signaling, prior to NF-kB activation, and the promotion of pro-inflammatory cytokines is illustrated in Fig. 8 (h). The current investigation has identified that MYD88 mRNA expression in the disease group (G II) showed a remarkable increase compared to the normal control group (G I). This points to a significant level of activation of innate immune signaling. It has been demonstrated that MYD88-dependent signaling is pivotal to the inflammatory cascade as indicated by the elevated levels of NF-kB, TNF-α, IL-1β, and IL-6 proteins. The substantial decline of MYD88 expression in G III, G IV, and G V demonstrates that the administered medication effectively suppresses the transcriptional inflammatory signal(s) at the upstream level. In particular, the highest treatment group (G V) showed that MYD88 expression approached normal levels indicating that TLR-mediated inflammatory activation was reset. This decrease in MYD88 expression accounts for the decreased cytokine burden and better inflammatory profile, as revealed by the biochemical assessments.

Fig. 8(i) shows TXNIP, the redox sensitive gene which serves as a critical nexus of oxidative stress, inflammasome activation, and metabolic dysregulation. The sickness group (G II) shows greater oxidative stress as demonstrated by an increase in MDA levels and IL-1β. The noted decrease in TXNIP expression in all treatment groups points to a suppression of oxidative stress signaling that is likely dose-dependent. Therefore, the drug appears to restore balance in the redox status (*i.e.* redox homeostasis), thus preventing the activation of TXNIP-dependent inflammatory pathways, such as the NLRP3 inflammasome. TXNIP expression in G V was, however, slightly higher than in the control group, which indicates an impressive enhancement of the cellular antioxidant defense. These transcriptional findings are evidence of reduced lipid peroxidation and improved oxidative status.

Fig. 8 (j) depicts BCL-2 expression and the control of apoptosis. BCL-2 is a notable anti-apoptotic gene that maintains the integrity of mitochondria and the cell's survival. Different from MYD88 and TXNIP, there was a notable drop in the expression of BCL-2 in the disease group, which points to the fact that there is a greater likelihood of cell undergoing apoptosis when there is chronic inflammation and oxidative stress. There is a notable increase in the expression of BCL-2 in the treatment groups, especially in G IV and G V, which states that the drug offers a degree of protection against the apoptosis that is brought about by inflammation. The elevation of BCL-2 expression is indicative of the improved cellular viability and integrity of the mitochondria which is consistent with the lowered expression of the causes of inflammation and oxidative stress. The inverse relationship between the reduced expression of TXNIP and elevated expression of BCL-2 indicates the controlled interactions of apoptosis with oxidative stress in the model. The sick state is signaled by elevation of MYD88, which starts inflammatory signaling with TXNIP is increased, which adds to oxidative stress and inflammatory, and BCL-2 downregulation which increases apoptosis sensitivity. This is an example of coordinated transcriptional control, and it foreshadows the changes in cytokines, markers of oxidative stress, and indicators of inflammation. The changes in the markers of inflammation and oxidative stress and the increased signaling of cell survival show that therapy preserves the state of the cells. It is important to target the MYD88/TXNIP and BCL-2 regulatory axes to achieve the changes that are observed in inflammation and stress protection.

### 4.7 Histopathological study

**Fig. 9:**
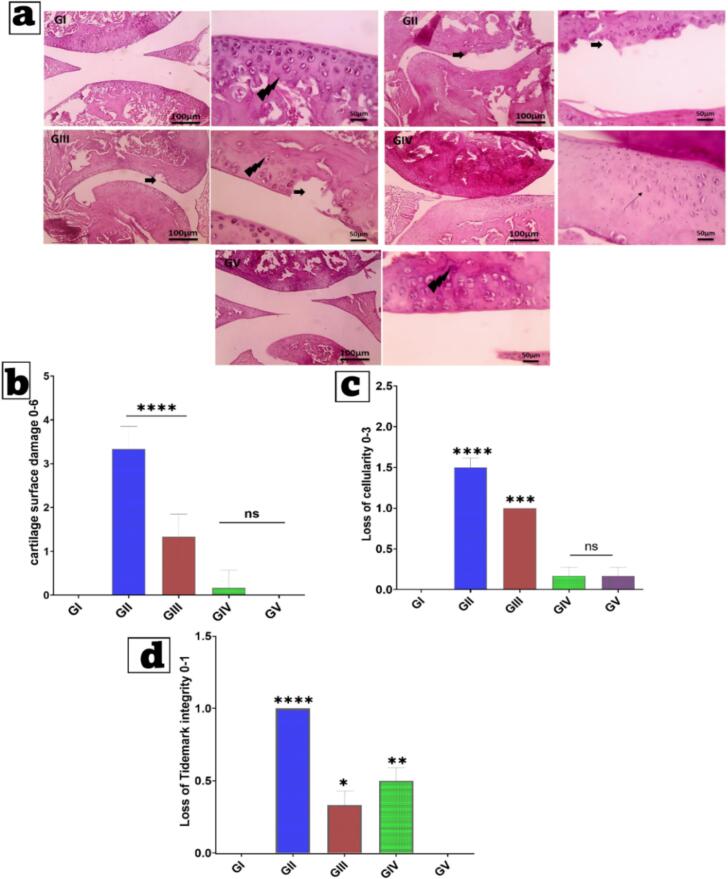
H&E histological assessment (a) and quantitative scoring of cartilage surface damage (b), cellularity loss (c), and tidemark integrity (d) in CFA-induced RA rats treated GI (healthy negative control), GII (RA model, untreated), GIII (treated with Naringin gel containing 2% w/w HPMC), GIV (treated with Naringin-loaded terpesomes in gel with 2% w/w HPMC), and GV (treated with Naringin-loaded terpesomes incorporated in Hyaluronic acid microneedles). H&E, X:100 scale bar 100 μm & X:400 bar 50 μm. Data are presented as mean ± SD (n = 10). *p < 0.05, **p < 0.01, ***p < 0.001, ****p < 0.0001 *versus* the negative.

Histopathological assessments were performed on the H&*E*-stained micrographs of knee joint sections for the cartilage retention and efficacy of the treatment formulations. The healthy control group (GI) showed the typical design of articular cartilage with a smooth superficial layer, organized cartilage matrix, and rounded chondrocytes in their respective lacunae. Furthermore, there were clear tidemarks differentiating non-calcified with calcified cartilage, as well composed zonation and structural zonation of the cartilage. In contrast, significant pathologies were revealed in the rheumatoid arthritis model group (GII). There were considerable superficial degradations at the articular cartilage, characterized by distinctly irregular, and fibrillated surfaces. Furthermore, there were chondrocyte dropouts, and decreased cartilage thickness with matrix structure compromise. In several regions, the tidal mark was absent or unclear, indicating the presence of degenerative rheumatoid arthritis-related joint damage. The findings correspond with the elevated Mankin scores in GII (Fig. 9b, c) depicting significant structural damage and deterioration of cartilage integrity. Application of NRG gel (GIII) resulted in quantifiable improvements including reduction in superficial cartilage erosion and restoration of tidal mark as shown in Fig. 9d. Chondrocytes moderate loss was also accompanied by a more organized surface of the articular cartilage compared to GII. This is indicative of some therapeutic effect. The Mankin score was significantly lower than the untreated RA group which also supports this observation. In the group that received NRG-TPs gel (GIV), more remarkable recovery was observed. The surfaces of the cartilage felt smooth. There was also a marked increase in the rounded viable chondrocytes that were in the defined lacunar spaces. Clearance and consistency of the tidal mark indicated restoration of zonal organization of the cartilage. The improvements observed histologically corresponds with lower Mankin scores which shows the terpesomal nanocarrier system's superiority to NRG gel. Compared to NRG-TPs/HA-MNs group (GV), the Mankin scores was the lowest and the most improved articular cartilage (Akanksha et al., 2025; ELhabal et al., 2024). There was a smooth surface with rounded chondrocytes that were well preserved and uniformly distributed in the cartilage matrix and the tidal mark was well visible in all the fields assessed showing the cartilage structure was thoroughly preserved and there were no degenerative changes. As was anticipated, GV revealed the most Mankin scores of all the treated cohorts, almost reaching the baseline value of the uninjured control. This finding corroborates the significant chondroprotective and restorative properties of microneedle-based delivery of terpesomes. In Fig. 9, we see a distinct therapeutic hierarchy. GV (NRG-TPs/HA-MNs) is superior to GIV (NRG-TPs gel) who is superior to GIII (NRG gel) and GII (untreated RA) as the worst. The changes between the groups demonstrate the increased efficacy of the joint targeting, soft tissue through the enhancement of microneedle-activated terpesome delivery, and the prolonged protective effect against the degeneration of rheumatoid arthritis (RA) affected cartilage.

### 4.8 Immunohistochemical (IHC) Examination of Transforming Growth Factor Beta (TGF-β)

**Fig. 10:**
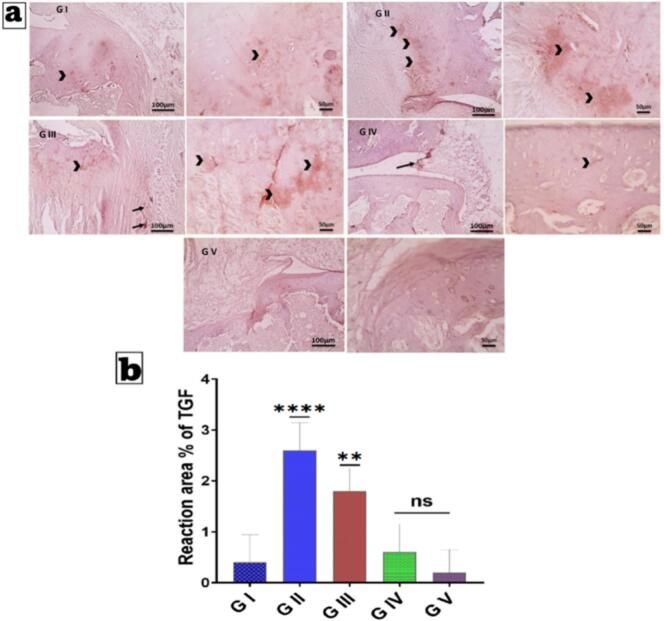
(a)Immunohistochemical photomicrographs of lung sections stained for Transforming Growth Factor-Beta (TGF-β) expression in different groups. Severe positive TGF-β expression is indicated in neoplastic cells (arrows). Group assignments: GI (healthy negative control), GII (RA model, untreated), GIII (treated with Naringin gel containing 2% w/w HPMC), GIV (treated with Naringin-loaded terpesomes in gel with 2% w/w HPMC), and GV (treated with Naringin-loaded terpesomes incorporated in Hyaluronic acid microneedles). (b) Quantitative analysis of TGF-β immunoreactivity, presented as reaction area percentage (%) IHC counterstained with Mayer's hematoxylin—low magnification X: 100 bar 100 μ and high magnification 400 bar 50 μ. Data are expressed as mean ± SD (n = 10). **p < 0.01, ****p < 0.0001 *versus* the negative control group.

Transforming Growth Factor-Beta (TGF-β) is a diverse cytokine involved in several biological processes including immune response and tissue repair. TGF-β is pivotal in cell proliferation, extracellular matrix formation, and immune cell restraint. In the context of rheumatoid arthritis (RA), TGF-β is pathologically dysregulated and contributes to the destructive processes of the disease. Fig. 10a and 10 b show immunohistochemical analysis of TGF-β expression among experimental groups, which reflects the extent of inflammatory response suppression achieved by each group (Choi et al., 2009; Tang et al., 2022). TGF-β staining in healthy controls (GI) shows normal tissue homeostasis. TGF-β staining in unmedicated RA (GII) shows robust immunostaining in articulating cartilage and peri-articular tissues which is indicative of unregulated TGF-β activity. Active RA is associated with TGF-β driven increases in inflammatory tissue (pannus), fibroblasts, and constituents of the extracellular matrix. After treatment with NRG gel (GIII), TGF-β staining showed Variation. In The cartilage, staining intensity was the same as the pre-treatment level while there was a slight reduction of staining in peri-articular tissues, which attests to the gel's anti-inflammatory effect. Rats administered NRG-TPs gel (GIV) demonstrated little TGF-β staining in cartilage and minor staining peri-articular which indicate improved tissue penetration and alteration of inflammatory pathways by the terpesomal nanocarrier. NRG-TPs/HA-MN group (GV) showed less immunostaining intensity than the other treatment groups, which indicates the absence of TGF-β expression in cartilage and periarticular regions. The staining profile that is indicative of normal conditions shows improved anti-inflammatory efficiency of microneedle-assisted delivery. This is due to increased absorption through the skin, sustained release, and directed delivery to the joints. The data indicates a distinct therapeutic hierarchy, whereby NRG-TPs/HA-MNs demonstrated the greatest reduction in the rheumatoid arthritis-driving TGF-signal inflammatory pathways (Tang et al., 2022). The results indicate a distinct therapeutic hierarchy, whereby NRG-TPs/HA-MNs demonstrated the greatest reduction in the rheumatoid arthritis-driving TGF-signal inflammatory pathways.

## 5 Conclusion

The study shows the extensive efforts taken to resolve path biopharmaceutical issues within the transdermal delivery system to treat rheumatoid arthritis with naringin. The system with the best nanoscale characteristics, optimal drug loading capacity, superior colloidal stability, high tensile strength, and flexibility was produced using PEGylated, limonene-based terpesomal nanocarriers and dissolving hyaluronic acid microneedle patches. The HA-microneedle matrix facilitated uniform skin penetration and sufficient transdermal delivery while keeping the patients safe and comfortable. Compared to conventional formulations, the NRG-TPs/HA-MNs system was found to achieve better therapeutic outcomes *in vivo* using CFA-induced rheumatoid arthritis animal model. The treatment resulted in the reduction of paw swelling and joint swelling while the joint structure was preserved as observed from the X-ray images. The key inflammatory markers such as MDA, TNF-α, IL-1β, IL-6, NF-κB, and MMP-3 were significantly decreased. Reduced MDA levels are consistent with the decreased oxidative stress. Immunohistochemistry and TGF-β levels showed increased levels which suggest being beneficial to cartilage, repairing tissue and control the disease. The loss of synovial inflammation and the gain of joint inflammation were evident. Naringin-loaded terpesomal Hyaluronic acid microneedles patches provide a novel, simple, and comfortable tool for the treatment of rheumatoid arthritis. The platform improves both the bioavailability and the efficiency of naringin, as well as providing a flexible transdermal system which, with modification, can be used for other poorly soluble anti-inflammatory drugs, thus allowing chronic inflammatory diseases to be managed safely and more effectively over a longer term.

## CRediT authorship contribution statement

**Sammar Fathy Elhabal:** Writing – review & editing, Methodology, Conceptualization. **Ahmed Mohsen Elsaid Hamdan:** Writing – review & editing, Formal analysis, Data curation. **Mai S. Shoela:** Writing – review & editing, Methodology, Formal analysis, Data curation. **Suzan Awad AbdelGhany Morsy:** Writing – review & editing, Methodology, Formal analysis, Data curation. **Amal M. Elsharkawy:** Writing – original draft, Methodology, Formal analysis. **Rana Saad-eldin:** Writing – review & editing, Methodology, Formal analysis. **Shady Allam:** Writing – review & editing, Methodology, Formal analysis. **Tassneim M. Ewedah:** Writing – review & editing, Formal analysis, Data curation. **Marwa A. Fouad:** Writing – review & editing, Methodology, Formal analysis. **Mahitab Elsayed:** Writing – review & editing, Data curation. **Hanan Mohamed Abd Elmoneim:** Writing – review & editing, Formal analysis, Data curation. **Halah Tariq Albar:** Writing – review & editing, Formal analysis, Data curation. **Wedian Younis Abdelgawad:** Writing – review & editing, Formal analysis, Data curation. **Fatma E. Hassan:** Writing – original draft, Formal analysis, Data curation.

## Institutional review board statement

The animal study protocol was approved by the Research Ethics Committee, Faculty of Pharmacy, Cairo University (REC-FOPCU, protocol code PI 3999 and approval at 29 September 2025). Animal models are among the most important *in vivo* models for fundamental parameters, such as drug efficiency, safety, and toxicological studies, as pre-clinical data are necessary for translating into humans. The ARRIVE criteria for animal research were adhered to in using and handling the study's animals.

## Submission declaration and verification

The authors declare that this manuscript has not been published or communicated for publication elsewhere, either in part or in whole.

## Funding

This research received no external funding.

## Declaration of competing interest

The authors declare that they have no known competing financial interests or personal relationships that could have appeared to influence the work reported in this paper.

## Data Availability

Data will be made available on request.
